# Electrochemiluminescence with semiconductor (nano)materials

**DOI:** 10.1039/d1sc06987j

**Published:** 2022-01-28

**Authors:** Yiran Zhao, Laurent Bouffier, Guobao Xu, Gabriel Loget, Neso Sojic

**Affiliations:** Univ Rennes, CNRS, ISCR (Institut des Sciences Chimiques de Rennes)-UMR6226 Rennes F-35000 France gabriel.loget@univ-rennes1.fr; University of Bordeaux, Bordeaux INP, ISM, UMR CNRS 5255 Pessac 33607 France neso.sojic@enscbp.fr; State Key Laboratory of Electroanalytical Chemistry, Changchun Institute of Applied Chemistry, Chinese Academy of Sciences Changchun P. R. China; University of Science and Technology of China Hefei Anhui 230026 China; Department of Chemistry, South Ural State University Chelyabinsk 454080 Russian Federation

## Abstract

Electrochemiluminescence (ECL) is the light production triggered by reactions at the electrode surface. Its intrinsic features based on a dual electrochemical/photophysical nature have made it an attractive and powerful method across diverse fields in applied and fundamental research. Herein, we review the combination of ECL with semiconductor (SC) materials presenting various typical dimensions and structures, which has opened new uses of ECL and offered exciting opportunities for (bio)sensing and imaging. In particular, we highlight this particularly rich domain at the interface between photoelectrochemistry, SC material chemistry and analytical chemistry. After an introduction to the ECL and SC fundamentals, we gather the recent advances with representative examples of new strategies to generate ECL in original configurations. Indeed, bulk SC can be used as electrode materials with unusual ECL properties or light-addressable systems. At the nanoscale, the SC nanocrystals or quantum dots (QDs) constitute excellent bright ECL nano-emitters with tuneable emission wavelengths and remarkable stability. Finally, the challenges and future prospects are discussed for the design of new detection strategies in (bio)analytical chemistry, light-addressable systems, imaging or infrared devices.

## Abstract 2

The combination of electrochemiluminescence and semiconductor gives rise to a rich field at the interface of photoelectrochemistry, materials and analytical chemistry. It offers interesting possibilities for ultrasensitive (bio)detection, imaging and light conversion.

## Graphical abstract

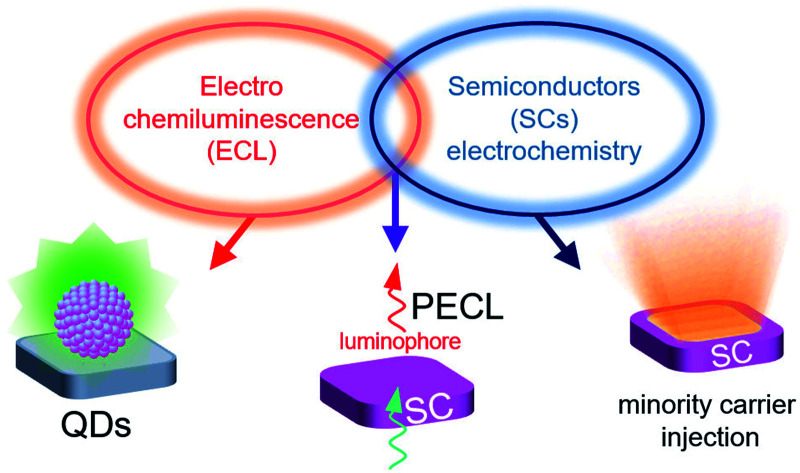

## 1. Introduction

Electrochemiluminescence (ECL), also called electrogenerated chemiluminescence, is the luminescence produced by electrode reactions.^1–5^ The different forms of these light-emitting electrochemical processes have interested a broad spectrum of the scientific community in the last decades.^6,7^ It started with the electrochemical generation of aromatic hydrocarbon radical ions and their recombination.^8,9^ It continued with the study of the famous Marcus “inverted” region since ECL is a direct verification of its occurrence.^10^ With the development of aqueous ECL based on sacrificial co-reactants,^2^ it has evolved progressively into a powerful analytical technique with many applications in (bio)analysis, environmental detection, light-emitting devices, and imaging.^4,11–13^ Particularly, ECL is successfully commercialized in clinical diagnostics with a large number of immunoassays, such as cardiac diseases, thyroid, infections, tumour markers, *etc.* Indeed, ECL technology does not require any external light source to photo-excite the luminophores as in fluorescence or phosphorescence. Therefore, it provides remarkable advantages such as very low optical background noise, high sensitivity, wide dynamic range, temporal and spatial controls of the ECL process by electrochemistry, and simplicity of the optical setup. Many works have been carried out to expand the capabilities of ECL, which still mainly relies in water on model luminophores such as luminol with H_2_O_2_ or [Ru(bpy)_3_]^2+^ with tripropylamine (TPrA) as co-reactant. However, in the above-mentioned application fields, there is an increasing demand for even higher sensitivity, multiplexing, stability, and portability.^6^ To enable greater ECL efficiency^14^ and brighter ECL (nano)-emitters with tuneable emission colours, many works have been carried out with original materials and novel detection schemes.^1,15–17^

With the continuous rise of semiconductor (SC) (nano)materials science and its miniaturization, the combination of ECL with SC materials offers the possibility to design ECL readout strategies with improved performances. For example, the field of SC photoelectrochemistry is extremely active for solar energy conversion, analytical chemistry, and spatially resolved photoelectrochemistry. In fact, photoelectrochemistry at illuminated SCs can be considered as the reverse interconversion process in comparison to ECL because light is converted into charge carriers. In other words, both involve the interplay between light and electrochemistry but the transformation of light into electrons follows opposite directions. Combining both phenomena (ECL and SC photoelectrochemistry) offers new possibilities for the detection schemes since electrodes can act as the light absorber and, at the same time, as the emitter. Moreover, a remarkable decrease of the electrochemical potentials required to generate ECL emission may be achieved. Among the different SCs, silicon is particularly attractive as a photoelectrode material because of its abundance, low toxicity, and tuneable electronic properties. However, it is highly unstable when used in oxidation in aqueous media and thus its passivation limits its applications in ECL. Indeed, in water, the majority of the ECL systems operates in oxidation and follows an oxidative-reduction mechanism with sacrificial co-reactant species (*vide infra*). Therefore, it implies generally the deposition of nanometer-thick protective layers over the Si material for its use in ECL.

The development of nanomaterials has opened new perspectives for ECL generation.^18–21^ In particular, switching from bulk to nanometric size of the SC modifies deeply the features of the materials. Indeed, the photophysical and electrochemical properties of a SC material are independent of its typical dimensions until it falls below a critical size, typically in the order of 10 nm. Down to this size regime, some SCs start exhibiting distinct physical characteristics compared to their “bulk” counterpart. This size effect has an impressive implication for optical transitions, which become size-dependent below a characteristic dimension for the SC. Such a behavior is a direct consequence of the spatial confinement of charge carriers in the SC crystal and it is generally referred to as “quantum size effect”.^22–24^ In a first approximation, the structure of colloidal luminescent SC nanocrystals, known as quantum dots (QDs), can be separated in 2 regions: the core (bulk phase) and its outer surface. Since such nano-objects exhibit a very large surface-to-volume ratio, the surface state energy levels related to the surface atoms differ from the bandgaps of the cores. This structural dichotomy is revealed by absorption and photoluminescence spectroscopies, which probe the contribution originating from the core part whereas electrochemical techniques probe mainly the surface. Since the surface states present generally lower energy levels than those from the core, ECL emission from the surface states is red-shifted in comparison to photoluminescence emission. QDs provide many useful features with a remarkable tuning of the electronic, electrochemical and optical properties resulting from the variations of their band electronic structures with respect to their size. Again, this is directly related to the quantum confinement effect. QDs possess high molar extinction coefficients, adjustable bandgaps, high photoluminescence quantum yields, and excellent photo- and chemical stabilities. Bard and coworkers investigated the electrochemistry of a series of QDs and the ECL emission using both annihilation and co-reactant modes.^25–28^ These seminal works opened new avenues to study the ECL emission from various QDs (*e.g.*, with groups II–VI, III–V, and IV–VI SCs) with different dimensions, structures, and shapes.^19,29,30^

Herein, we review the recent advances showing how ECL technology benefits from the SC materials starting from the bulk materials and down to the nanoscale. After presenting briefly the ECL mechanisms, we introduce the concepts and practical considerations of the photoelectrochemical charge-transfer processes occurring at SC surfaces. In Section 4, the SCs are used as the electrode materials and we discuss advances in ECL at the surface of bulk SCs, especially through the photogeneration of minority carriers at the surface of various n- and p-type photoelectrodes. In Section 5, the size of the SC materials is decreased and the resulting QDs are bright ECL nano-emitters as illustrated with selected examples for (bio)sensing. In brief, we describe how SC materials with different sizes and designs of the experiments may behave as the ECL-emitting electrodes themselves, as the photo-activated electrodes to induce ECL in solution or, more classically, as the ECL emitters in solution. Finally, the conclusion section presents challenges and outlooks of this field.

## 2. ECL mechanistic paths

ECL is the light emission produced by electrode reactions.^5^ It belongs to the family of luminescence phenomena but with an electrochemical component. This broad definition of ECL approved by IUPAC covers a large spectrum of light-emitting processes occurring at the electrode surface.^5^ It is noteworthy that ECL has been defined by the IUPAC commission on photochemistry and not on electrochemistry. ECL can be produced through 3 main mechanisms: (i) homogeneous exergonic electron-transfer reactions involving the luminophore (*e.g.*, [Ru(bpy)_3_]^2+^) usually dissolved in solution; (ii) bond-breaking reactions within the luminophore frames (*e.g.*, luminol) and (iii) a special route called hot electron-induced ECL (HECL). In the first case, the luminophore is regenerated during the ECL process and may emit a photon many times whereas it is irreversibly consumed in the second path because it involves a dissociative bond-breaking or atom-transfer step. It means that along this path (ii) each luminophore will emit a photon just once during the ECL process. The first two pathways are the most widely used. On the contrary, HECL shares some specific features with electroluminescence (*i.e.*, radiative recombination of electrons and holes in a material, usually a SC).

### 2.1. Homogeneous exergonic electron-transfer reactions

In this pathway, the excited state of the luminophore is generated by an exergonic electron-transfer reaction between two species (or two redox states of the same species). They recombine in solution and a fraction of the luminophore involved reaches its excited state. This recombination reaction lies in the inverted Marcus region.^10^ Indeed, the Marcus theory on the rates of electron-transfer reactions predicts two regions: the normal one where the reaction rate increases with the free enthalpy of the reaction and the inverted region where kinetic inhibition occurs with increasing exergonicity of the reaction.^31^ ECL lies in the inverted region because the formation of the ground state is kinetically inhibited in favour of the excited state.^32^ Two main sub-pathways, namely the annihilation and co-reactant (implying a sacrificial species named co-reactant) paths can be distinguished.^7^

In the annihilation process, oxidized A˙^+^ and reduced B˙^−^ forms of an entity (an atom, a molecule, a complex, a nanoparticle, a QD, *etc.*) are produced at the electrode surface either by pulsing sequentially the electrode potential at cathodic and anodic values or at two separated electrodes operating in reduction and oxidation.^33–35^ Then these two species react together according to an annihilation reaction involving the exchange of an electron and generate the excited state of the luminophore. A and B can be the same molecular entity or nanoparticle but with different redox states. In the case of a QD, the sequence of steps leading to the annihilation process is:

1 QD → QD˙^+^ + e^−^ (oxidation at the electrode)

2 QD + e^−^ → QD˙^−^ (reduction at the electrode)

3 QD˙^−^ + QD˙^+^ → *ε* QD* + (2 − *ε*) QD (electron-transfer reaction)

4 QD* → QD + *hν* (emission of the ECL photon)

where *ε* reflects the efficiency of the excited state generation.

This simple route implies that the electrogenerated species can keep their redox states long enough to annihilate upon colliding with oppositely charged species. For bioanalytical applications, the potential window of aqueous solutions is usually not wide enough to form these stable reduced and oxidized species. Thus, modern ECL applications follow a different mechanistic route involving a sacrificial co-reactant.

A co-reactant is a chemical reagent that is either oxidized or reduced generally at the electrode surface.^7^ The resulting species generate very reactive radicals, which exchange an electron with the oxidized or reduced luminophore and populate the desired excited state. The corresponding mechanisms are referred to as “oxidative-reduction” and “reductive-oxidation” ECL, respectively. The role of the co-reactant is to provide strong reducing or oxidizing radicals that will generate the excited state by an exergonic electron-transfer reaction with the oxidized or reduced luminophore.^36,37^ During the process, the co-reactant is consumed irreversibly whereas the luminophore is regenerated and can participate in a new ECL cycle. Therefore, an excess of co-reactant is usually employed. Typical anodic co-reactants following the “oxidative-reduction” pathway are oxalate, TPrA, NADH, 2-(dibutylamino)ethanol (DBAE) and they are widely used in aqueous solutions.^38,39^ Persulfate and benzoyl peroxide (BPO) are typical cathodic co-reactants operating in organic solvents.^7^ For TPrA, the mechanism involving a QD can be written as:^40^

5 QD → QD˙^+^ + e^−^ (oxidation at the electrode)

6 TPrA → TPrA˙^+^ + e^−^ (oxidation at the electrode)

7 TPrA˙^+^ → TPrA˙ + H^+^ (deprotonation)

8 QD˙^+^ + TPrA˙ → *ε* QD* + (1 − *ε*) QD + P (electron-transfer reaction)

9 QD* → QD + *hν* (emission of the ECL photon)

where P is a byproduct of TPrA during the ECL process.

### 2.2. Bond-breaking reactions within the luminophore frame

Bond-breaking reactions or atom-transfer reactions within the luminophore frame itself may populate the excited state. But, the following energetic criterion should therefore be fulfilled: this chemical reaction has to be sufficiently energetic to deliver enough free energy to generate the excited state. Luminol and its derivatives, especially L-012 (8-amino-5-chloro-2,3-dihydro-7-phenyl-pyrido[3,4-*d*]pyridazine-1,4-dione),^41,42^ are the most common ECL compounds following this mechanistic pathway.^4^ At basic pH, oxidation of luminol at the electrode surface produces a diazaquinone intermediate, which reacts with hydrogen peroxide. Thus, it generates 3-aminophthalate in an excited state due to an O–O bond cleavage in the endoperoxide form. Finally, it emits a characteristic blue light. Luminol ECL has been applied to measure either luminol or species labelled with luminol or peroxides.^43^

### 2.3. Hot electron-induced ECL pathway

HECL involves “hot electrons” (*i.e.*, energetic electrons having higher energy than the Fermi level of the surrounding phase) that are injected into an aqueous electrolyte solution from oxide-covered electrode surfaces.^44,45^ It is a special pathway in ECL, which employs cathodically pulse-polarised thin insulating film-coated electrodes such as oxide-covered aluminium or silicon electrodes. These electrodes behave as cathodic surfaces due to the strong reducing capability of the injected hot electrons. The tunnel injection of hydrated electrons leads to the formation of oxidizing radicals (*e.g.*, sulphate radicals) from added co-reactants. The produced strong oxidants and reductants react with solute luminophores that emit light. In this context, a large variety of luminophores (*e.g.*, Tb(iii) chelates, organic fluorophores, luminol and [Ru(bpy)_3_]^2+^) have been reported and used for bioassays.^46–48^

## 3. Semiconductors and their photoelectrochemistry

### 3.1. Bulk semiconductors

**Fig. 1:**
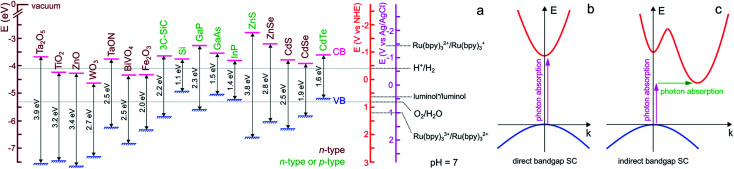
(a) Scheme showing the positions of the VB and CB edges of selected SCs and their bandgap values, as well as the standard potential of selected ox/red couples at pH 7. n-type SCs are indicated in brown and SCs that can easily be made n- or p-type are indicated in green. The band edge values were taken from ref. 54 except for BiVO_4_,^55^ Fe_2_O_3_,^52^ and 3C-SiC,^56^ that were taken from the indicated refs. For the oxides, a Nernstian behavior of the electrolyte band edge with pH was assumed. The standard potentials involved in ECL reactions were extracted from ref. 57–59. Note that actual bandgap values may vary by approximately ±0.2 eV. (b) Scheme of electron energy *vs.* wave vector showing the optical transition in a direct bandgap SC (b) and an indirect bandgap SC (c). In the latter case, the assistance of a phonon is required to grant the transition.

For ease of reading of the following sections, we now briefly summarize the well-established fundamentals of SC materials. Inorganic SC materials are often classified into groups defined by the column of their atoms in the periodic table. Thus, elemental SCs such as Si or Ge are referred to as group IV (now group 14 of the periodic table) whereas numerous compound SCs such as binary or ternary alloys belong to group III–V (*e.g.*, GaP), or group II–VI (*e.g.*, CdSe). Since many transition metal-based materials are also SCs, they are also often classified by their chemical composition, for instance, binary transition oxides (*e.g.*, TiO_2_) or oxynitrides (*e.g.*, TaON). SCs having a long-range periodicity such as single crystals (*e.g.*, Czochralski-grown Si) or epitaxial films are commonly used in photoelectrochemical applications and often lead to excellent photoconversion efficiencies.^49,50^ However, their preparation methods can be considered prohibitive. Consequently, polycrystalline materials manufactured by simpler wet methods (*e.g.*, electrodeposition, electroless deposition, hydrothermal, or anodization)^51^ are sometimes preferred in applications for which material cost is a key limitation.^52,53^ SCs can have a wide variety of crystal structures, however, most SCs from groups IV and III–V adopt diamond and zinc blende structures, respectively. Compared with metal or insulator materials, the uniqueness of SC materials lies in their band structure, which originates from their crystalline organization. In materials, electronic states corresponding to orbitals are distributed in energy bands that can be populated by electrons. At the equilibrium, the highest filled energy band is referred to as the valence band (VB) and the lowest vacant energy band is referred to as the conduction band (CB). While the VB and CB of group IV SCs involve bonding and antibonding sp^3^ outer orbitals, the case of compound SCs is different due to the high polarity of their bonds. In TiO_2_, for instance, the VB is composed of oxygen 2p orbitals and the CB by the Titane 3d orbitals. One of the key parameters of a SC is its bandgap that is defined as the energy gap between the highest edge of the VB and the lowest edge of the CB and its value is referred to as *E*_g_. As shown in Fig. 1a, in SCs, *E*_g_ is typically comprised between 0.5 and 4 eV.

Optical transitions of electrons from the VB to the CB are effective through the absorption of photons of an energy higher than *E*_g_. The nature of the bandgap, which is defined by the band structure of the crystal, can be direct or indirect. As illustrated in Fig. 1b, in direct bandgap-SCs (*e.g.*, amorphous Si (a-Si)), the transitions are fully allowed while in indirect bandgap-SCs (*e.g.*, crystalline Si (c-Si)), a simultaneous lattice vibration (a phonon) is required to allow photon absorption, which decreases the absorption probability (Fig. 1c). This distinction is consequently reflected by a large difference in the absorption coefficient (*α*), thus, to absorb a given density of incident photons, an indirect-bandgap SC must be thicker than a direct-bandgap SC. For this reason, the c-Si wafers employed in photovoltaic solar cells have a thickness of a few hundreds of μm while sub-μm-thick a-Si layers are typically employed in low power devices such as solar-powered calculators. Optical transitions through the bandgap result in the creation of electron/hole e^−^/h^+^ pairs. A free electron is promoted in the CB leaving an h^+^ (an electronic vacancy) in the VB. After e^−^/h^+^ generation and charge generation, an important aspect is the transport of these charge carriers through the material. The conductivity of undoped (also called intrinsic) SCs is too low to promote efficient charge transport, thus, to be employed in light-conversion applications. This is the reason why SCs are generally doped by atoms referred to as shallow donors and acceptors. Shallow dopants are ionized at ambient temperature and improve conductivity by promoting free e^−^ or h^+^ in the CB or the VB, respectively. The dopant concentration controls the concentration of free carriers and the dopant nature (donor or acceptor) defines the majority carriers in the SCs (e^−^ or h^+^). SCs having CB electrons as majority carriers are referred to as n-type and SCs having VB holes as majority carriers are referred to as p-type (the doping type of several SCs is indicated in Fig. 1a with a color code). Besides affecting their conductivity, doping also influences another important energetic parameter, that is, the Fermi level (*E*_f_). This parameter is the electrochemical potential of a phase, and, for a solid phase, it is defined as the energy where the probability of occupation by an electron equals ½. *E*_f_ is located exactly in the middle of the bandgap in an intrinsic SC whereas *E*_f_ is located just below the CB edge in an n-type SC (n-SC) that has been moderately doped with donor atoms. Conversely, *E*_f_ is located just above the VB edge in a p-type SC (p-SC) that has been moderately doped with acceptor atoms. The Fermi level allows correlating the SC electronic properties with those of the contacting phase (*e.g.*, a metal, another SC, or an electrolyte).

### 3.2. Semiconductor quantum dots: size matters at the nanoscale

**Fig. 2:**
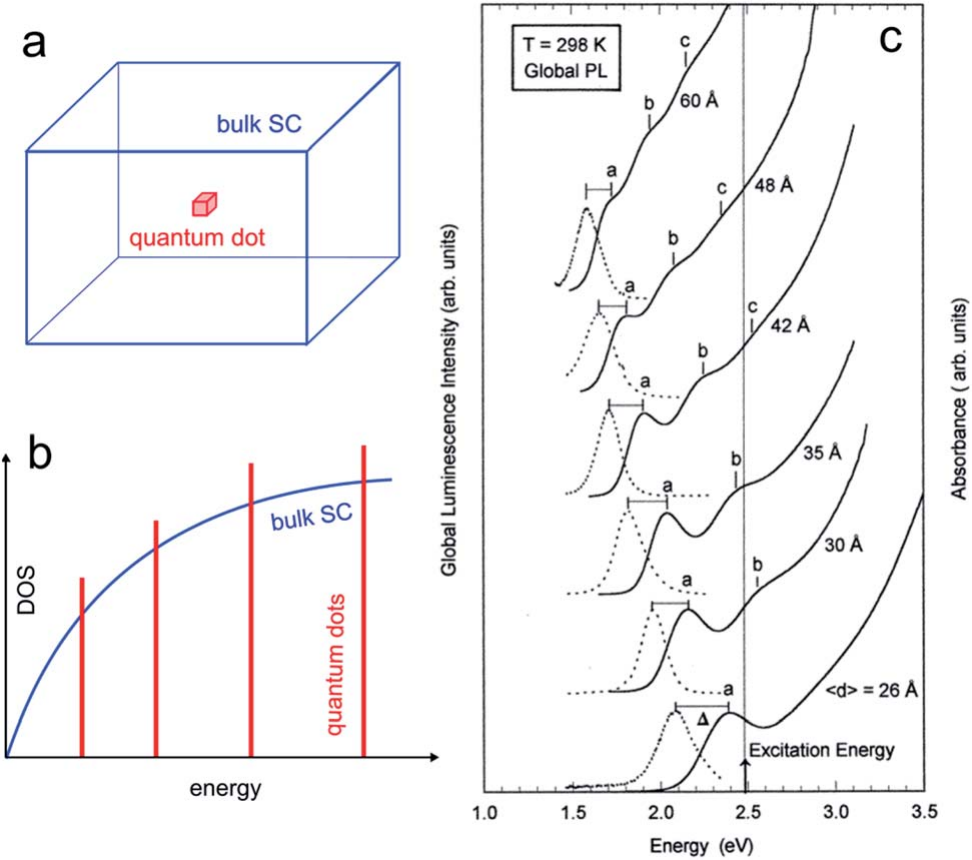
(a) Scheme showing a 3D bulk SC (blue) and a “0D” quantum dot (red). (b) Density of states (DOS) functions for a bulk SC (blue) and a quantum dot (red). (c) Absorption (solid line) and global PL (dotted line) spectra for InP quantum dots with different mean diameters. The photoexcitation was done at 2.48 eV. Reproduced from ref. 74 with permission from the American Chemical Society, Copyright 1997.

The properties of SC material are independent of the material size until it falls below a critical dimension.^60–62^ Indeed, quantum mechanics predicts a continuous-to-discrete transition of the density of states when the dimension of SC particles is similar to or lower than the exciton Bohr radius (the characteristic distance between the electron and the hole), as shown in Fig. 2a and b. Quantum confinement also often results in a shift of the bandgap value as a function of size, and typically, *E*_g_ increases for smaller SC particles.^63,64^

The quantum size effect can be encountered in particles of 2 dimensions (2D, quantum wells), 1D (quantum wires), and 0D, as illustrated in Fig. 2a and b. The latter case, namely, 0D particles exhibiting quantum size effect is of particular interest in this article, and such particles are generally referred to as QDs. They typically consist of SC nanocrystals having a size in the range of ≈10 nm and below. The critical size where the quantum size effect occurs depends on the effective masses of the charge carriers, and, because this parameter is strongly dependent on the SC material, not all SCs can exhibit this effect.^65,66^ As an example, Fig. 2c illustrates how the size of InP QDs affects their photoluminescence (PL).

From an historical perspective, A. Ekimov discovered QDs in the early 1980s by synthesizing copper halides (CuCl, CuBr) and cadmium chalcogenides (CdS, CdSe) nanocrystals in a molten glass matrix.^67^ Their unprecedented optical behavior was explained by the confinement of their electrons according to the theoretician A. Efros. At the same period, the chemist L. E. Brus proposed a colloidal route to QDs that eases their handling and opens the door to practical applications.^68^ Based on these pioneering works, QDs physical chemistry has been developed over four decades and is now vastly reported in the literature. QDs show promise for a large number of applications including nanoelectronics, solar cells, and medicine.^69–71^

In a seminal report, Z. Ding *et al.* described the electrochemistry of Si QDs with the reversible electrochemical injection of discrete numbers of electrons.^25^ Moreover, they produced ECL upon electron and/or hole transfer. Rapidly, they also showed the possibility to generate ECL from CdSe QDs with a substantially red-shifted emission from the PL spectrum.^27^ This opened the door to the exploration of a large variety of SC materials, such as Ge, CdTe, CdSe, and PbSe (see Section 5). However, the chemical reactivity of the QD surface may generate traps for charge carriers and thus it leads to a low ECL efficiency. The difference observed between PL and ECL spectra illustrates the different mechanisms operating in PL and ECL, as well as in electroluminescence.^72^ Therefore, specific criteria for the fabrication of highly efficient QDs should be taken into account to avoid surface traps for electrons and/or holes.^72,73^

### 3.3. Photoelectrochemistry at semiconductors

Photoelectrochemistry at SCs has a long history. The first photoelectrochemical experiments were performed in 1839 by E. Becquerel who discovered a photovoltaic effect at an illuminated AgCl electrode.^75^ The so-called “Becquerel effect” was understood in the 1950s^76^ and, in the early 1970s (a period marked by the oil crisis), a breakthrough article by Fujishima and Honda reporting the photoelectrochemical water electrolysis using a TiO_2_ photoanode sparked the popularity for photoelectrochemistry at SCs.^77^ In this section, we briefly introduce the thermodynamic basics of the SC/liquid junction and we will present how such interfaces can be employed beneficially in the frame of photoelectrochemistry. We will only focus on the description of a bulk SC electrode phase, a topic that has been covered in several articles and books to which we refer the interested reader.^78–81^ We note, in passing, that similar effects can also occur for other SC/phase junctions such as for a SC in contact with another SC (*e.g*., in a p–n junction) or a SC in contact with metals (*e.g*., in a Schottky junction). While all these junctions can be used in photoelectrochemistry,^82,83^ for the sake of simplicity, we only discuss here the classical SC/liquid junction.

**Fig. 3:**
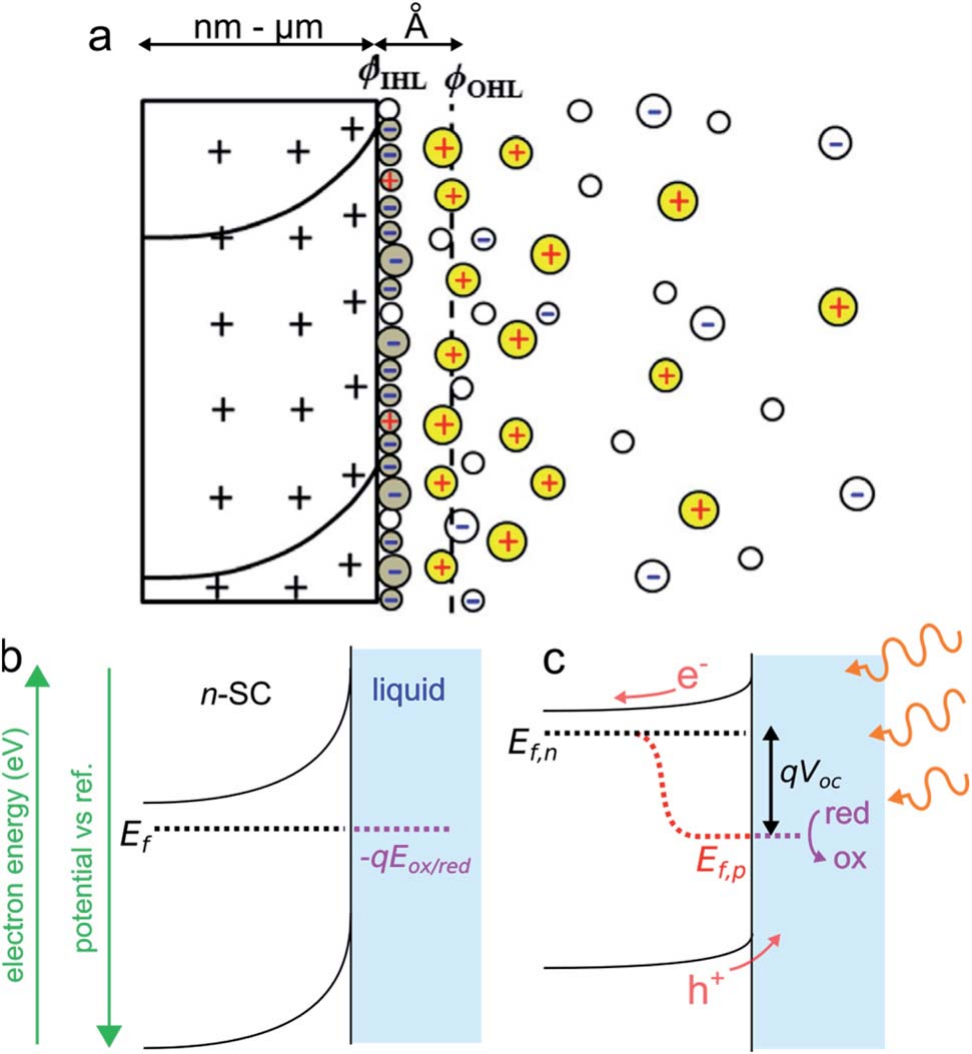
(a) Scheme of the space charge layer in an n-SC (left) and the Helmholtz layer at the SC/liquid interface (right), adapted with permission from ref. 92*ϕ*_IHL_ and *ϕ*_OHL_ are the inner and the outer Helmholtz planes. (b) Scheme showing a depleted n-SC in equilibrium with an electrolyte containing the ox/red redox couple in the dark. (b) Scheme showing the effect of illumination (*hν* > *E*_g_) on this junction.

A typical n-SC/liquid interface at equilibrium is shown in the simplified scheme of Fig. 3a. This interface is composed of several layers that are: (i) the space charge layer in the outermost part of the SC (thickness of 10 nm to several μm), (ii) the Helmholtz layer (thickness <1 nm), and (iii) the diffuse layer (thickness of ≈10 nm).^84^ When a SC is brought in contact with an electrolyte containing a dissolved ox/red redox couple, electrostatic equilibrium must be attained and their electrochemical potential (*i.e.*, their Fermi level) must become equal. This occurs *via* charge transfer through the phases, which will lead to a variation of *E*_f_ in the SC because the solution has a higher density of states.^81^ In the case of an n-SC equilibrating with a solution having an electrochemical potential −*qE*_ox/red_ (in eV) located below its *E*_f_, charges will transfer from the SC (that becomes positively charged) to the liquid phase (which becomes negatively charged) until equilibrium is reached. This phenomenon creates a distribution of uncompensated ionized dopants within the SC (which becomes positively charged in the case of an n-SC, as shown in Fig. 3a). Conversely, equilibration of a p-SC with a solution having an electrochemical potential located above its *E*_f_ would lead to a distribution of negative charges within the SC (not shown here). This situation strongly differs from the situation encountered in a metal where the charge is located at its surface. This leads to a region fully depleted of free carriers that is referred to as the space charge layer (SCL) or the depletion region (Fig. 3a). The width of the SCL, *w*, is related to the dopant density and is typically in the order of 10 to 1000 nm. In the case of a depleted n-SC, the positive charge in the SCL induces a decrease in the CB and VB energy within the SC that remains flat outside of the SCL. This phenomenon is referred to as band bending and is the origin of the so-called built-in electric field. Note that an upward band bending is represented in Fig. 3b, but a downward band bending would be the result of an opposite scenario, *i.e.*, a p-SC in equilibrium with an electrolyte with −*qE*_ox/red_ > *E*_f_. As discussed and shown in Fig. 3b, *E*_f_ ideally equilibrates with −*qE*_ox/red_, however, band bending can also be strongly affected by the composition of the interface. Often, the presence of surface states control band bending through Fermi level pinning.^85^ Interestingly, dynamic electrostatic phenomena occurring at the interface can also alter the junction energetics.^86^ The built-in electric field within the SCL has important consequences, as it will direct the flux of charge carriers when charge separation occurs in the SCL. Indeed, in the situation depicted in Fig. 3b, h^+^ (which are not represented here because the junction is in the dark) would be directed toward the liquid phase while electron would be directed toward the bulk of the SC. Electrochemical reactions at the SC interface are affected by the density of charge carriers at the interface, which is dependent on the applied potential and the illumination. In the dark, charge transfer processes involving species in solution are dominated by the majority carriers. As a consequence, depleted n-SCs can carry out reductions but not oxidations, since no h^+^ are present to oxidize the reductant species (this is the opposite for depleted p-SCs that can only trigger oxidation reactions in the dark due to the lack of available electrons). Therefore, in the dark, depleted SCs can be considered as rectifying diodes allowing only one type of faradaic process. The situation when the SCL is illuminated with incident light having an energy *hν* > *E*_g_ (*ν* is the frequency of light) is very different from that described previously. Indeed, under such conditions, photogenerated e^−^/h^+^ pairs are separated by the electric field present in the SCL. The light source can be of different nature. If it is typically a laser, a light-emitting diode, or an arc lamp, it has been shown that photoeffects at SC electrodes can be obtained from excited states in solutions, generated through chemiluminescence^87–89^ or ECL.^90,91^

In the case shown in Fig. 3c, the electrons are driven inside the SC and the h^+^ are directed at the solid/liquid interface where they can participate in oxidation reactions. Thus, illumination of n-SC promotes photoelectrochemical oxidation (conversely, illumination of p-SC promotes photoelectrochemical reduction). In this out-of-equilibrium situation, the charge carrier density, as well as the band bending, is altered within the SC and the carrier populations can no longer be described by a single Fermi level. It is, therefore, possible to describe the SC energetics using the concept of separate quasi-Fermi levels for each band, that is the h^+^ quasi-Fermi level, *E*_f,p_, and the electron quasi-Fermi level, *E*_f,n_ (Fig. 3c). The electrochemical potential of the illuminated SC/liquid interface becomes thus the quasi-Fermi level of its minority carrier, that is, *E*_f,p_. It is crucial to note that, under illumination, the electrochemical potential of the interface (*E*_f,p_) has lower energy than that of the bulk SC (*E*_f,n_), thus, electrochemically speaking, the potential at the interface of the illuminated junction is more positive (more oxidative) than the bulk SC. The difference between these two energies is referred to as photovoltage *V*_oc_. To sum up, the photovoltage is a consequence of the energy mismatch between the SC and the liquid contacting phase. It is a specific phenomenon of photoactive junctions that allows triggering electrochemical reactions with a lower potential than that of conductive electrodes, and these illuminated interfaces are referred to as photoelectrodes. Generally speaking, illuminated n-SCs promote oxidation reactions at a lower potential than conductive electrodes and are called photoanodes, and, illuminated p-SCs promote reduction reactions at a higher potential than conductive electrodes and are called photocathodes. Practically speaking, *V*_oc_ values and band diagrams of the SC/liquid junction can be extracted based on experimental electrochemical measurements.^79,93^

Photoelectrochemistry at illuminated SCs is still an intense topic of research, with a prominent activity in the field of solar energy conversion since photoelectrochemical cells can be seen as a potential technological solution for reducing carbon emissions through H_2_ production or CO_2_ valorization.^94,95^ Furthermore, other research activities in photoelectrochemistry at SCs are developed in the frame of analytical chemistry since photocurrent can be employed as a detection signal for chemical analysis.^96–98^ Of particular interest, the tunability of the incident excitation pattern allows SC photoelectrodes to be employed for spatially resolved photoelectrochemistry.^99,100^ Thanks to the progress made on the stabilization of SC/liquid interfaces over the last decade (see next section), this field of research has experienced a recent regain of interest in recent years^101^ as it opens enticing opportunities in the fields of sensing, biology, and surface patterning.^102–105^

### 3.4. Semiconductor photoelectrode designs

A major concern of SC photoelectrodes is their stability as most SCs are subject to degradation through corrosion or photocorrosion (when used under illumination), which leads to a massive degradation of the electrochemical performances.^106^ The origin of this fading of activity is due to competitive electrochemical reactions occurring at the photoelectrode surface during their utilization. Either photocathodes (p-SC) or photoanodes (n-SC) are prone to such a deleterious instability when their self-reduction potential and self-oxidation potential are located above and below the potential at which they are used, respectively. In general, photocorrosion is more pronounced for photoanodes than for photocathodes. Several n-type metal oxides, such as TiO_2_ or WO_3_, have high chemical stability,^107^ however, these materials suffer from low charge mobility and their wide bandgap restricts their spectral absorption. All photoelectrodes able to provide a broad spectral absorption are highly sensitive to photocorrosion. For instance, Si passivates directly upon immersion in aqueous solutions or exposure to air with the spontaneous formation of insulating native surface oxide that grows further when operated in the anodic regime. The problem of photocorrosion has been a bottleneck for the conception of photoelectrochemical cells for decades and is still the subject of intense research.^108^ This matter can be particularly problematic in the frame of ECL that is based on highly exergonic reactions that require the application of large anodic or cathodic potentials (see Section 2). Several strategies employed so far imply the introduction of an additional conformal protection layer at the SC surface in almost all cases.^106,108^ To design an efficient protection layer, drastic considerations must be taken into account. First, the coating should allow a charge flow through its intrinsic conductivity or by tunneling, it should not deteriorate the energetics of the solid/liquid interface and it should not impede light absorption. Metal conformal thin films have been employed to protect photoelectrodes with the great benefit of being able to generate a Schottky junction at the SC/metal interface when the metal work function is adapted to the *E*_f_ of the SC, thus promoting charge extraction. On Si-based photoanodes, a material that is particularly known for its instability in the anodic regime, it has been shown that inhomogeneous metal coatings such as randomly dispersed metal nanoparticles prepared by aqueous electrodeposition (an easy and cost-effective method), can efficiently promote photogenerated charge transfer at the SC/liquid interface,^109,110^ allowing stability for water oxidation in alkaline media up to several days. In this case, the passive SiO_*x*_ layer generated during anodic polarization protects Si from alkaline dissolution. Metal oxide or chalcogenide thin layers^111–113^ can also be used as protective layers, and most research has been currently focused on TiO_2_ conformal films of different natures,^114,115^ due to their chemical inertness, good electronic properties, and low price. Another protection strategy, which has been shown versatile in aqueous media uses a monolayer of alkyne-terminated alkyls (1,8-nonadiyne) covalently attached on the Si surface.^116^ This approach is particularly interesting because it allows grafting molecular moieties on the monolayer through “click” chemistry in addition to avoiding photocorrosion of the SC.

Another interesting aspect of photoelectrode design is surface micro- and nanostructuration. From an optical point of view, improved light absorption can be induced for nano- and microstructured SC by multiple internal reflections or a smooth transition of refractive index, respectively.^117–119^ Besides, the main advantage of using a structured photoelectrode compared to a planar is that it allows decoupling light absorption and carrier collection.^120^ For instance, for SCs suffering from a short minority carrier diffusion length like oxides, using a structure with features smaller than the diffusion length (typically ≈5 nm for h^+^ in α-Fe_2_O_3_) can decrease recombination losses. However, care must be taken when using micro- and nanostructured photoelectrodes as they often also imply disadvantages compared to their planar counterparts, including the absence of band bending in features with a size smaller than the SCL, the presence of a higher density of recombination sites at their surface, a decreased photovoltage caused by the mismatch between the geometric and the projected area of the SC and complex mass transport phenomena.^121,122^

## 4. ECL at the surface of bulk semiconductors

### 4.1. Degenerate semiconductors

In Section 4, we will present cases for which ECL emission occurs at the planar surface of bulk SCs (*e.g.*, wafers), or structured SCs (*e.g.*, porous Si prepared by electrochemical etching).^123^ In this subsection, we will briefly discuss the particular case of SCs whose doping is so high that their electrical behavior is similar to that of conductors. These SCs are referred to as degenerate and their Fermi level is located above/below the conduction/valence band edges for n- and p-types, respectively. Electrochemically, they exhibit no photoactivity and essentially behave as “classical” electrodes such as glassy carbon or gold, although they may exhibit different reactivity due to their intrinsic chemical composition. Typical degenerate SCs that commonly serve as ECL substrates are transparent conducting oxides (TCOs), such as indium tin oxide (ITO) and fluoride-doped tin oxide (FTO) thin films. They provide unique optical properties and their transparency is often crucial for recording ECL emission, for instance, in microscopy applications or thin-film luminescent devices.^124^ Other SCs materials such as Si can be intentionally heavily doped. Degenerate Si is referred to as p^+^-Si or n^+^-Si, depending on the doping type. In principle, efficient and stable ECL from molecular systems can be achieved in the dark at degenerate SC surfaces, provided that it is protected or resistant enough towards corrosion (see Subsection 3.4). An interesting application of ECL is that it can be used to investigate electrical passivation resulting from degradation, which is often a crucial issue. Indeed, the growth of a passive oxide layer on an operating electrode is expected to result in ECL fading. This concept has been recently explored in a bipolar electrochemical configuration,^125,126^ with the [Ru(bpy)_3_]^2+^/TPrA system, using degenerate Si bands or squares patterned in microfluidic channels.^127–129^ Degenerate SCs are also used in the area of HECL,^45,130–134^ a specific field of ECL research in which an insulating oxide serves as a filter for hot electrons (electron of high thermal energy) generated in a conducting electrode (see Subsection 2.3). Owing to the planarity of Si and its ability to be conformably covered by its insulating oxide, n^+^-Si and p^+^-Si have been used to manufacture electrodes for HECL that were employed in immunoassays.^134–136^

### 4.2. Emission from the semiconductor through minority carrier injection

Luminescence can be induced by minority carrier injection in SCs. In the archetypal case, an oxidant with a sufficiently high standard redox potential injects an h^+^ in the VB of a n-SC, which recombines with the majority carrier, generating photons.^79^

**Fig. 4:**
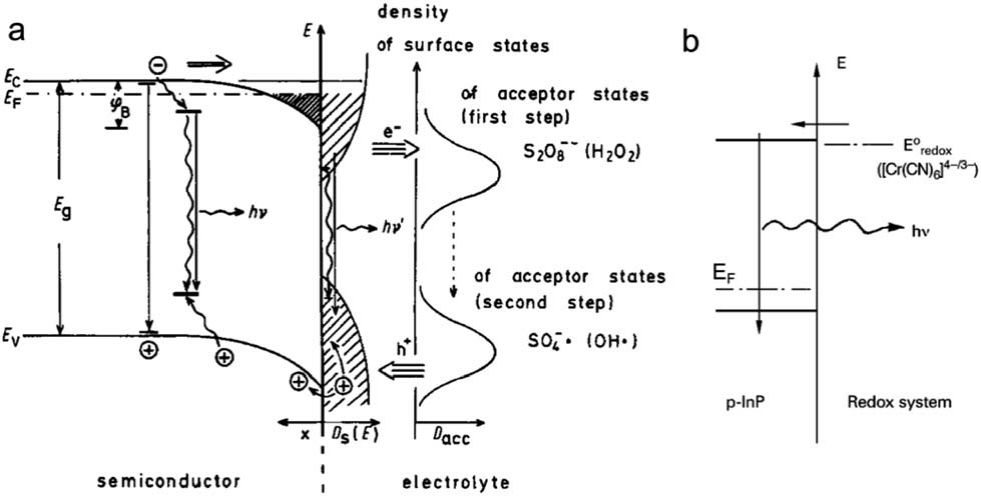
(a) Scheme showing the ECL mechanism at the surface of a n-SC, where the cathodically-generated radical injects a hole in the VB of the SC. Reprinted with permission from ref. 137. (b) Electron injection into the CB of p-InP and recombination. Adapted from ref. 79 with permission from Wiley-VCH, Copyright 2015.

This phenomenon has been extensively studied in the eighties and was formerly referred to as electroluminescence. However, given the definition of ECL set in the introduction, it can be also considered as ECL because an interfacial electrochemical reaction is required to trigger the emission at the SC. A peculiarity in this process is that unlike in classical ECL systems based on dissolved luminophores (see Section 2), the emitter is, here, the solid SC surface. In this situation, the SC is operated in the accumulation regime and electrochemistry occurs in the dark. Many studies concerned planar n-SCs in the cathodic regime, among which: GaP, GaAs, InP, CdS, ZnS, SnO_2_, ZnO, TiO_2_, Ta_2_O_5_, and ZnGa_2_O_4_.^137–142^ These studies were performed in aqueous or organic media with dissolved oxidants such as Fe(CN)_6_^3−^, Ce^4+^, or aromatic compounds.^138,143^ An alternative reliable method to generate emission, which is depicted in Fig. 4a, consists in using a dissolved reactant that, following the electrochemical reduction, generates a highly reactive oxidant at the vicinity of the n-SC, leading to h^+^ injection. Typical reactants are S_2_O_8_^2−^, H_2_O_2_, and other compounds such as NO_3_^−^ and HOCl.^137^ Whereas many emissive systems based on h^+^ injection at n-type SCs have been reported, much fewer examples involved electron injection in the CB of p-SCs. The rare examples involved electron injection from Cr(CN)_6_^4−^ (Fig. 4b) or methyl viologen cation radical in the CB of p-InP.^79,144^ In another study, emission was observed during the anodic dissolution of p-InP, it was thus concluded that, during this process, a decomposition intermediate injected electron in the CB.^145^ This type of ECL, and the comparison of its spectral characteristics with the PL can be very useful for the fundamental investigation of SC/liquid interfaces. It can be used for investigating reaction mechanisms,^146^ and can also be employed for sensing.^147^

A particular SC material that must be discussed independently is porous Si. The luminescence of porous Si has been the topic of intense research.^123,148^ Specifically, the first ECL observation was reported in 1960 ^149^ and it has attracted considerable interest in the nineties.^150^ Porous Si can be easily produced by simple chemical and electrochemical methods, allowing the preparation of porous n-Si or p-Si layers with tuneable pore size on commercial wafers. Porous Si exhibits strong PL, with spectral characteristics very different from its planar counterpart, which exhibits very weak PL due to its indirect bandgap. It has been admitted that emission from nanosized crystallites in the porous layer is affected by the quantum size effect (see Section 3.2). For that reason, porous Si exhibits enhanced luminescence as well as widening of its bandgap, controlled by the size of the nanosized Si crystallites, which, in turn, affects the shape of the luminescence spectrum.

**Fig. 5:**
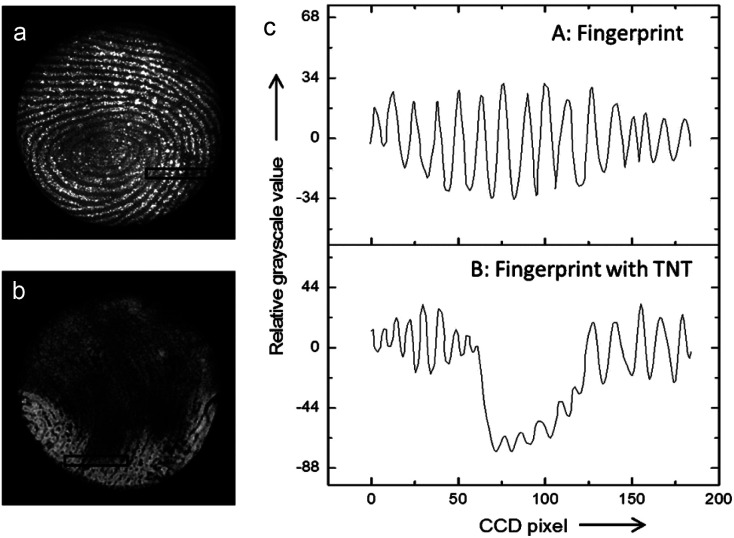
(a and b) Picture obtained by an ECL-based image-contrast technique showing (a) the image of a latent fingerprint on porous Si, (b) the latent fingerprint impressed with the same fingertip pretreated with TNT. (c) Cross-sectional relative grey scale value over 15 parallel ridges within the area indicated by the black rectangles in (a) and (b). Reproduced from ref. 143 with permission from Wiley-VCH, Copyright 2014.

Interestingly, it has been shown that the ECL mechanisms based on minority carrier injection, discussed above for planar SCs, are also effective on porous Si,^151^ and they generally lead to intense luminescence in the visible region. Moreover, porous Si can emit by many other mechanisms (including chemiluminescence and photoinduced ECL).^152,153^ Among other applications,^154^ the ECL of porous Si has been employed for detection. An illustrating example is shown in Fig. 5. In this work, Su and coworkers have shown that the ECL of porous p-Si, generated upon e^−^ injection can reveal latent fingerprints and that ECL can be quenched if trinitrotoluene (TNT) was present on the finger.^143^ Later, the authors extended their approach to image the fingerprints on ITO.^155^ More recently, the ECL of n-type nanostructured TiO_2_ nanotubes or ZnO nanorods,^156^ prepared by anodization of metal plates or hydrothermal methods has attracted interest.^157,158^ In a similar manner as that previously discussed for planar n-SCs, these substrates exhibited cathodic ECL in the presence of O_2_, S_2_O_8_^2−^ and H_2_O_2_, with different spectral features^156,159–162^ compared to their planar counterpart.^163^ TiO_2_ nanotubes have been loaded with CdS nanocrystals to develop an ECL assay for the detection of prostate-specific antigen (PSA).^164^

### 4.3. Emission from a molecular luminophore in the dark

Another method to produce ECL in the dark at the surface of SCs is to use an electrolyte containing a molecular ECL system (such as the ones presented in Section 2) and generate the excited state by a majority carrier process. This situation differs from that described in Subsection 4.1 because, here, the electrode has SC properties, and the benefit of this approach compared with that described in Subsection 4.2 is that it allows using well-known efficient ECL systems and can lead to a more intense and more stable ECL emission. In this approach, of course, the instability of the SC under polarization (Section 3.4) may still be an issue, therefore, n-type SCs operating in the cathodic regime are preferentially used. In the late seventies, a series of works was performed to determine if excited states could be produced from a direct heterogeneous charge transfer^165–167^ to an oxidized species. This fundamentally differs from the typical annihilation case where the oxidized and the reduced species react in the homogeneous phase to produce ECL (see Section 2). In these studies, electrolytes containing the oxidized form of a luminophore (R˙^+^) were employed for annihilation studies at the surface of n-SCs. If the formation of an excited state is very unlikely to occur by a direct charge transfer at the surface of a metal electrode, it was predicted that the formation of the excited state could be allowed for a wide bandgap SC if the standard potential of R˙^+^/R lies in the gap and if the excited state level is located close to the flat-band potential. In these conditions, electron charge transfer from the CB would likely populate the excited state level, leading, thus, to direct ECL. This concept was tested for different luminophores and wide bandgap n-type SCs.^165,166,168^ ECL could be indeed observed at a more positive potential than that required to generate R˙^−^ on a few systems with a transient time controlled by the lifetime of the excited state. Direct electron transfer to produce an excited state was demonstrated for oxidized rubrene at n-ZnO and n-CdS in carefully purified organic solvents^165,168^ and for [Ru(bpy)_3_]^3+^ at n-SiC and n-GaP in organic and aqueous electrolytes.^167^ A similar concept was also employed with a metal electrode (Ta) covered by an n-type oxide (Ta_2_O_5_) with cation radicals or with [Ru(bpy)_3_]^3+^ for demonstrating that hot electrons^169^ can be injected into the solution phase *via* the CB.^170,171^

### 4.4. Photoinduced ECL (PECL)

**Fig. 6:**
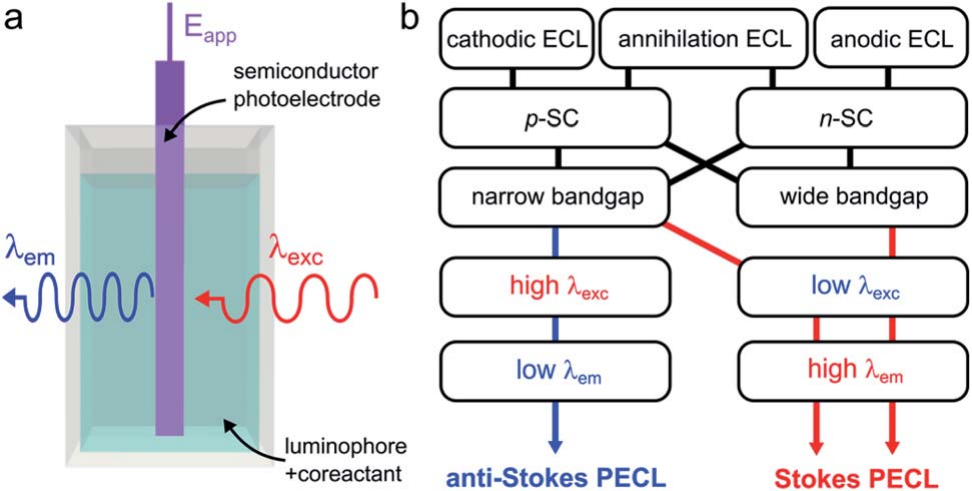
(a) Scheme showing light conversion by PECL. (b) Scheme showing the possible combinations of ECL systems and SCs, leading to the two PECL categories.

Photoinduced ECL, referred to as PECL, is a process in which an excited state is generated by photogenerated minority carriers at the surface of a SC. It is therefore a process triggered under illumination, most of the time, with a luminophore dissolved in the solution phase. In PECL, the SC photoelectrode absorbs incident light at a given excitation wavelength *λ*_exc_ and the ECL reaction at the solid/liquid interface generates light at another emission wavelength, *λ*_em_, as shown in Fig. 6a. PECL is thus a light conversion process where the intrinsic optical and electrochemical properties of the SC absorber and the ECL system emitter are used synergistically.

PECL requires an illumination as well as applying a potential to occur and cannot be generated if only one of these two stimuli is applied alone. A characteristic of PECL is that it allows triggering ECL at a lower (absolute value) applied anodic or cathodic potential (*E*_app_) than at a conducting electrode due to the photovoltage generation (see Section 3.3). Furthermore, this is a very versatile concept from an optical point-of-view, because the SC material, as well as the ECL system, can be selected to generate a specific conversion. The first main parameter is the SC bandgap, which dictates the upper limit for *λ*_exc_. Indeed, to yield absorption by the photoelectrode, the energy of the incident photons cannot be lower than the SC bandgap. The second important parameter is the luminophore employed since it controls the *λ*_em_ value. Although being fundamentally different by nature, several similarities can be noted between PECL and photoinduced chemiluminescence, in which a reaction triggered by UV absorption generates excited states.^172^

In PECL, several light conversion routes are possible and one can separate PECL processes into two main categories, depicted in Fig. 6b. The first one, which was first reported,^173^ results in the emission of photons of higher energy than that absorbed, *i.e.*, *λ*_exc_ > *λ*_em_, and can be seen as an electrochemically assisted upconversion process, we refer to this type as “anti-Stokes PECL”. The second category of PECL produces photons of lower energy than that absorbed, *i.e.*, *λ*_exc_ < *λ*_em_, like in classical PL processes, we refer to this second type as “Stokes PECL”. Because visible emission is generally desirable, anti-Stokes PECL can only be generated at narrow bandgap SCs, as they grant optical absorption until the IR. Conversely, Stokes PECL can be, in principle, indifferently obtained with wide or narrow bandgap SCs, since they both absorb low *λ*_exc_. Besides, PECL is possible through anodic and cathodic reactions. Another important aspect is the choice of the ECL system, which controls *λ*_em_, and, thus, the magnitude of the shift between *λ*_exc_ and *λ*_em_. Illustrating examples for the two PECL categories are summarized in Table 1. In the following, we review the PECL examples reported in the literature. To highlight the chronological aspect, we will start with the works performed in organic electrolytes, and, then, we will present more recent works achieved in aqueous electrolytes.

**Table 1:** Main characteristics of several reported PECL systems

PECL type	SC	ECL system/process	*E*_app_ a (V)	*λ*_exc_ (eV)	*λ*_em_ (eV)	*λ*_shift_ (nm)	Ref.
Anti-Stokes	p-Si	Annihilation	—b	1.7^c^	3	−316	173
	p-InP						
	p-GaAs						
Anti-Stokes	p,n-GaAs	Annihilation	1.6 V^d^	<1.7	2.8^e^	−293	176
	p,n-InP						
Anti-Stokes	n-Si	[Ru(bpy)_3_]^2+^/TPrA	0.5 V	1.5	1.9	−175	111
Anti-Stokes	n-Si	Luminol	−0.1 V	1.7^f^	2.9	−300	174
Stokes	n-BiVO_4_	L-012	−0.4 V	3.3	2.5	128	180
Stokes	n-Fe_2_O_3_	L-012	−0.2 V	3.3	2.5	123	179

a Potential onset of the PECL emission, *vs.* Ag/AgCl, if not reported otherwise.

b The reference electrode was not indicated.

c *λ*_exc_ = 674 nm (1.8 eV) was also reported.

d Difference between the anodic *E*_app_ and cathodic *E*_app_ for n-GaAs.

e For the TMPD/DPA system on n-GaAs.

f *λ*_exc_ = 625 nm (2 eV) was also reported.

#### 4.4.1. PECL in organic electrolytes

The first PECL experiments were performed through the annihilation mechanism (see Section 2) in a carefully dried organic solvent. Such media allow accessing a large electrochemical window, which is often required to generate the reactive radicals that will lead to the excited state. It also grants better stability of the SC material, which tends to degrade in the presence of air and water. The first evidence for PECL was reported in the mid-seventies,^173,175^ at the surface of narrow bandgap photocathodes of p-Si (*E*_g_ = 1.1 eV), p-InP (*E*_g_ = 1.3 eV), and p-GaAs (*E*_g_ = 1.4 eV) illuminated with near IR light (729 nm). The electrolyte was composed of 9,10-dichloro-9,10-dihydro-9,10-diphenyl-anthracene (DPACl_2_), dissolved in acetonitrile. Upon electrochemical reduction, the anion radical DPA˙^−^ chemically reacts with an intermediate to generate the DPA excited singlet that emits at 413 nm. PECL was triggered at the surface of the p-Si photocathode with a photovoltage of ≈300 mV. Interestingly, this article also introduced localized PECL generation, generated with the beam of a 674 nm laser.^173^ In this report, annihilation PECL was generated through a cathodic step (followed by a spontaneous chemical step).

A more “conventional” manner to trigger annihilation PECL is to generate the anion radical and the cation radical by applying a squarewave signal with sequential anodic and cathodic pulses (see Section 2). This route was explored for p- and n-type InP (*E*_g_ = 1.3 eV) and GaAs (*E*_g_ = 1.4 eV) illuminated above 729 nm with a series of organic compounds in acetonitrile.^176^ PECL was observed for all SCs at potential excursions (*i.e.*, the difference between the potential required to generate the anion radical and the potential required to form the cation radical) that were considerably reduced when compared to that required on (non-photoactive) Pt. It was mentioned that most emissions were unstable over time due to the competing degradation of the SC material (see Subsection 3.4). Nonetheless, it was possible to obtain stable blue PECL with *N*,*N*,*N*′,*N*′-tetramethyl *p*-phenylenediamine (TMPD)/9,10-diphenyl-anthracene (DPA) for n-GaAs and 10-methylpheno-thiazine (10-MP)/fluoranthene (FLU) for p-GaAs.^176^ For all PECL systems that were tested in this work, the two cited are the ones that required an oxidative excursion at the more negative potentials, corresponding thus to less corrosive conditions for GaAs.

#### 4.4.2. PECL in aqueous electrolytes

**Fig. 7:**
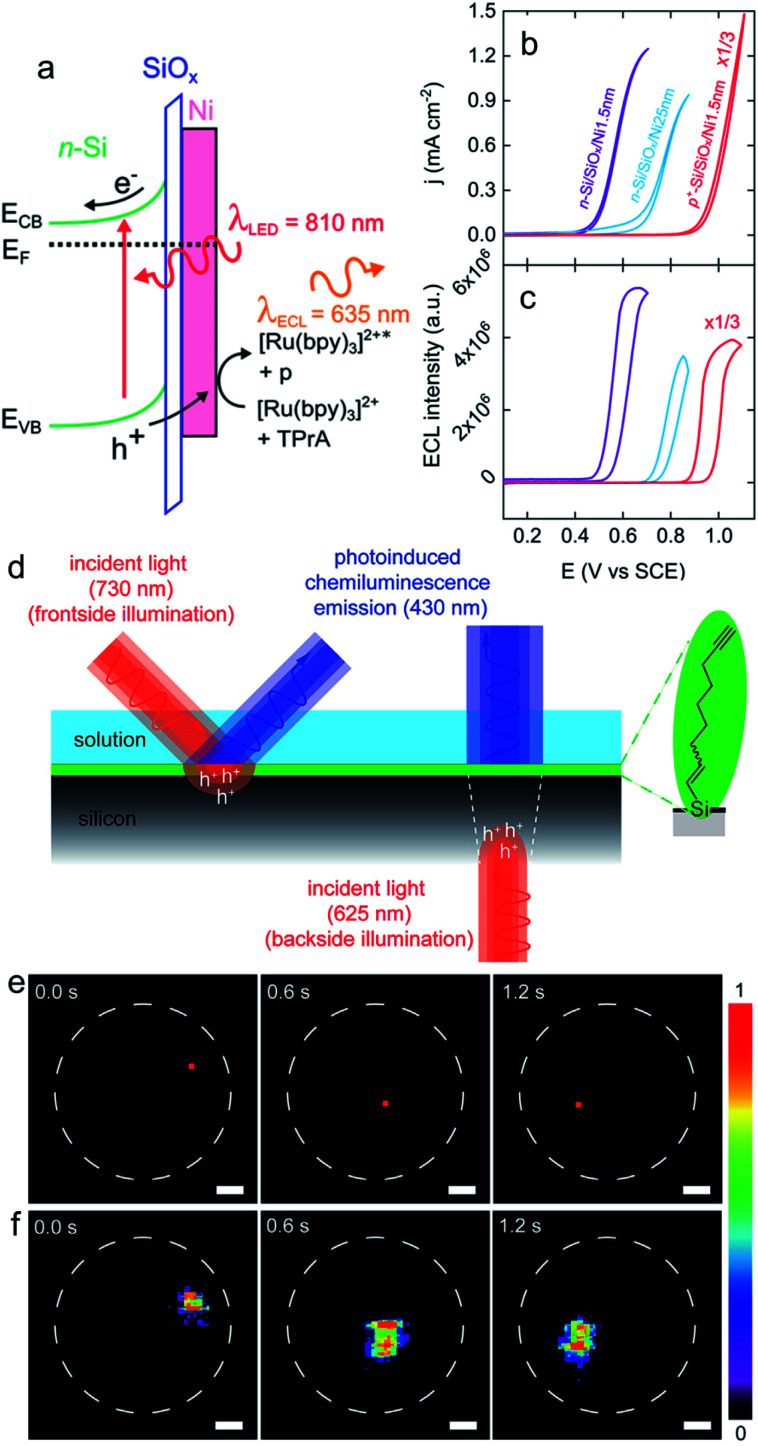
(a) Scheme showing a n-Si/SiO_*x*_/Ni (Metal–Insulator–Semiconductor, MIS) photoanode emitting anti-Stokes PECL (the side product of the TPrA radicals formed during the ECL process is denoted by p). (b) Cyclic voltammograms recorded in the dark on p^+^-Si/SiO_*x*_/Ni (thickness of the Ni thin film = 1.5 nm) (red curve) and under illumination on n-Si/SiO_*x*_/Ni (thickness of the Ni thin film = 1.5 nm) (purple curve) and n-Si/SiO_*x*_/Ni (thickness of the Ni thin film = 25 nm) (blue curve). (c) Corresponding ECL intensity *vs. E* curves. These measurements were performed in PBS (pH 7.3) with 5 mM [Ru(bpy)_3_]^2+^ and 100 mM TPrA at 20 mV s^−1^. Adapted from ref. 111 with permission from the American Chemical Society, Copyright 2019. (d) Schematics depicting the monolayer chemistry and the spatiotemporal control of PECL in either front (left) or backside (right) illumination mode. (e) A dynamic red-light pattern (730 nm, squares with side of 200 μm) illuminates sequentially specific areas of a macroscopic silicon-electrolyte interface. The dashed white circles indicate the limit of the interface. (f) Corresponding video frames (false-color intensity, intensity bar is in arbitrary units) showing the 2D mapping of the electrogenerated chemiluminescence emission. The electrolytic solution contained 0.1 M KOH, 0.05 M luminol, and 1% (v/v) H_2_O_2_. Electrode: n-Si(111) of low doping, modified with 1,8-nonadiyne, substrate illumination: frontside 730 nm, *E*_app_ = 0.5 V, scale bars: 1 mm. Adapted from ref. 174 with permission from Cell Press, Copyright 2020.

First, an interesting report of ECL amplification at glassy carbon, coupled with photocurrent generation, was reported.^59^ Even though this material is not generally employed for its semiconducting properties, it has been shown that its illumination above 500 nm increased the ECL of luminol (*λ*_em_ = 450 nm) occurring at 0.2 V *vs.* Ag/AgCl. The pH dependency of the process was reported. The authors suggested that under illumination, photogenerated hot h^+^ create OH˙ radicals from OH^−^, which react with luminol to produce more intense ECL. This explanation implies the photogeneration of highly energetic holes, since the standard potential of the OH˙/OH^−^ couple is 1.90 V *vs.* SHE.^177,178^ Due to the conducting nature of the working electrodes, such a mechanism is very different from the PECL occurring on classical SC photoelectrodes. This is further stressed by the fact that no shift of onset potential was measured upon illumination.^59^ The recent progress in SC photoelectrode stabilization^83,108^ (Section 3.4) allowed to develop PECL systems that can be operated in aqueous electrolytes, opening doors for the development of PECL with modern co-reactant systems. For instance, using a Ni thin film with a thickness in the nm-range sputtered onto a chemically oxidized Si surface (Si/SiO_*x*_) allows to preserve Si and to use this material for stable ECL (in the case of degenerate p^+^-Si) or PECL (in the case of n-Si) of the anodic [Ru(bpy)_3_]^2+^/TPrA co-reactant system^111^ (Fig. 7a). In this work, anti-Stokes PECL could be generated with *λ*_exc_ = 810 nm and *λ*_em_ = 635 nm, as shown in Fig. 7b and c. The operation of the photoanode was demonstrated for 15 min under galvanostatic control and the thickness of the Ni thin film has a strong effect on the photovoltage, with a maximum value of 410 mV.^111^ Later, the PECL of luminol/H_2_O_2_ at n-Si protected with a covalently grafted monolayer of alkyne-terminated alkyls was reported,^174^ as shown in Fig. 7d. In this work, PECL was triggered at a potential as low as −0.1 V *vs.* Ag/AgCl (photovoltage of ≈500 mV), with an excitation at 730 nm and emission at 430 nm. The possibility of performing spatially resolved PECL using a collimated beam was investigated and demonstrated with a frontside illumination at 730 nm (Fig. 7e and f) and a backside illumination at 625 nm on photoelectrodes thinned down to 70 μm. Due to the shorter diffusion of photogenerated minority carriers, the best resolution (sub-mm) was achieved with frontside illumination.^174^

**Fig. 8:**
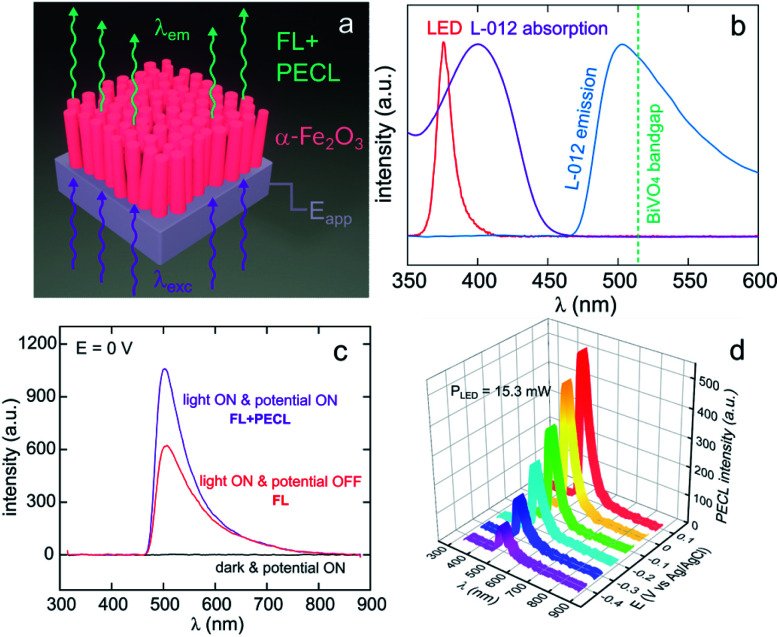
(a) Scheme illustrating the luminescence amplification principle based on the generation of Stokes PECL by the backside-illumination of an oxide-based photoanode (here, hematite). Reproduced from ref. 179 with permission from Elsevier, Copyright 2021. (b) Normalized spectra showing the emission of the LED used as the excitation source (red), L-012 absorption (purple), and the emission of L-012 (blue). The experimentally determined bandgap of BiVO_4_ is represented by the green dotted line. (c) Luminescence spectra recorded at an applied potential of 0 V in the dark (black curve), at open circuit under LED illumination (red curve, FL), and at 0 V under LED illumination (purple curve, FL + PECL). (d) PECL spectra recorded with a constant LED illumination at different potentials. The measurements were recorded with an illumination intensity of 15.3 mW (*λ*_exc_ = 375 nm) in 0.1 M NaOH with an L-012 concentration of 10 mM. Reproduced from ref. 179 with permission from Elsevier, Copyright 2021.

Lately, Stokes PECL was investigated at n-type oxides in water, as shown for hematite in Fig. 8a. These SC materials have better stability than narrow bandgap SCs and have been widely studied in the frame of photoelectrochemical water splitting. They have a wider bandgap than Si and some III–V SCs and can only absorb high-energy photons. In these works, the PECL of a luminol analog, 8-amino-5-chloro-2,3-dihydro-7-phenyl-pyrido[3,4-*d*]pyridazine-1,4-dione (L-012) was studied in alkaline electrolyte. The first example of Stokes PECL employed BiVO_4_, a n-SC having a bandgap of 2.4 eV, as a photoanode.^180^ The SC was deposited by electrodeposition and subsequent annealing onto FTO-coated glass slides. UV illumination (*λ*_em_ = 375 nm) was employed and it should be noted that L-012 absorbs at that wavelength too, producing fluorescence (Fig. 8b). The electrode was backside-illuminated (from the air/SiO_2_/FTO interface) which led to L-012 fluorescence at open-circuit conditions. When the potential is applied to a value where L-012 is oxidized, PECL occurs, which leads to the considerable amplification of the overall luminescence (fluorescence & PECL), as shown in Fig. 8c. Due to the high photovoltage at the BiVO_4_/liquid interface and the low oxidation potential of L-012, PECL was generated at a potential as low as −0.4 V *vs.* Ag/AgCl (Fig. 8b).^180^ The PECL emission was dependent on the illumination intensity, the applied potential, and was stable for ≈2 h under potentiostatic control.^180^ This PECL mechanism was also tested on another type of photoanode, made of nanostructured hematite (α-Fe_2_O_3_) nanorod arrays (Fig. 8a), deposited on FTO-covered glass by hydrothermal synthesis.^179^ The same photoelectrochemical investigation was performed and it was found that PECL could be generated at a more positive potential of −0.2 V *vs.* Ag/AgCl on this photoanode. Besides, if the electrode could be operated for ≈2 h, a degradation of the PECL intensity and the photocurrent density was recorded during prolonged operation.^179^

## 5. ECL of semiconductor quantum dots

### 5.1. Introduction to semiconductor quantum dots ECL

**Scheme 1:**
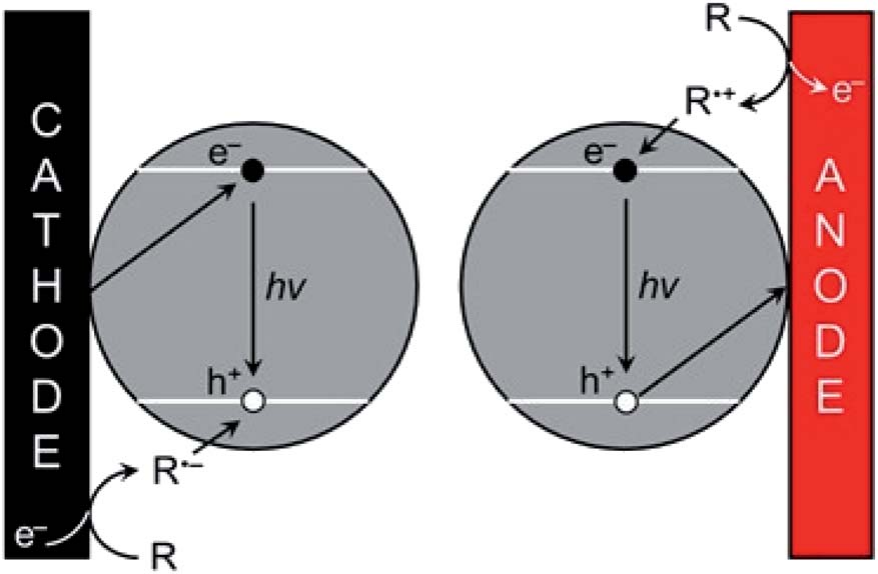
ECL promotion with QDs. The e^−^/h^+^ pairs are injected separately by the electrode and the co-reactant, respectively following either the cathodic (left) or anodic ECL pathway (right). Adapted from ref. 181 with permission from the American Chemical Society, Copyright 2014.

As explained in Section 3.2, the optical and electronic properties of QDs are extremely size-dependent. Photoexcitation of QDs leads to narrow PL with precise control of the emission wavelength depending on the size and structure of the QDs. However, the translation of PL properties to ECL capability appears challenging since the mechanism leading to the excited state generation is rather different. In PL mode, an exciton is simply generated by absorbing a photon. Practically, the hole is injected in the VB whereas the electron is injected in the CB. Therefore, their quick recombination within the QDs is not a problem contrarily to ECL mode. The ECL mechanism is initiated by heterogeneous electron-transfer steps that are promoted at the solid electrode/liquid electrolyte interface. The main ECL route involves the reaction of a luminophore (*i.e.*, a QD in the present case) with a sacrificial co-reactant (see Section 2). Both chemicals are simultaneously reduced or oxidized in order to promote a sequence of reactions leading to the excited state (Scheme 1).^181^ According to these mechanisms, the key step is the decomposition of the species electrogenerated from the co-reactant since this additional dissociative chemical step that follows the interfacial electron-transfer enables the production of a reductant following electrochemical oxidation or reversely an oxidant following an initial reduction at the electrode surface. However, it is essential to inject the electron and the hole (one originating from the electrode and the other one from the co-reactant transformation) separately in the QD. As a result, ECL generation from QDs is essentially controlled by the charge separation and therefore by surface traps. This is why the chemical engineering of the QDs architecture should ensure sequential e^−^ and h^+^ injections and possibly long-lasting ECL duration by comparison to PL or electroluminescence.

On the other hand, one essential reason that might also explain the ECL suitability of QDs is simply their very small size. Indeed, it is well-known that micro- and nanoscale (semi-)conducting objects such as nanoparticles, QDs or micro/nano-electrodes enable a very efficient mass transport regime, related to the dominance of radial diffusion,^182–184^ that increases markedly the resulting faradaic current. In the context of ECL, such property can substantially affect the overall efficiency either with colloidal QDs dispersed in the electrolyte or adsorbed at the electrode surface. In addition, regardless of the experimental configuration, the wiring between the electrode and the QDs might also become a key issue. Unfortunately, these very important aspects are consistently eluded in most reported works and a general effort towards a more precise description of the electron-transfer reaction at QDs is still required.

### 5.2. Historical groundbreaking reports in the 2000s and rationalization of the properties

**Fig. 9:**
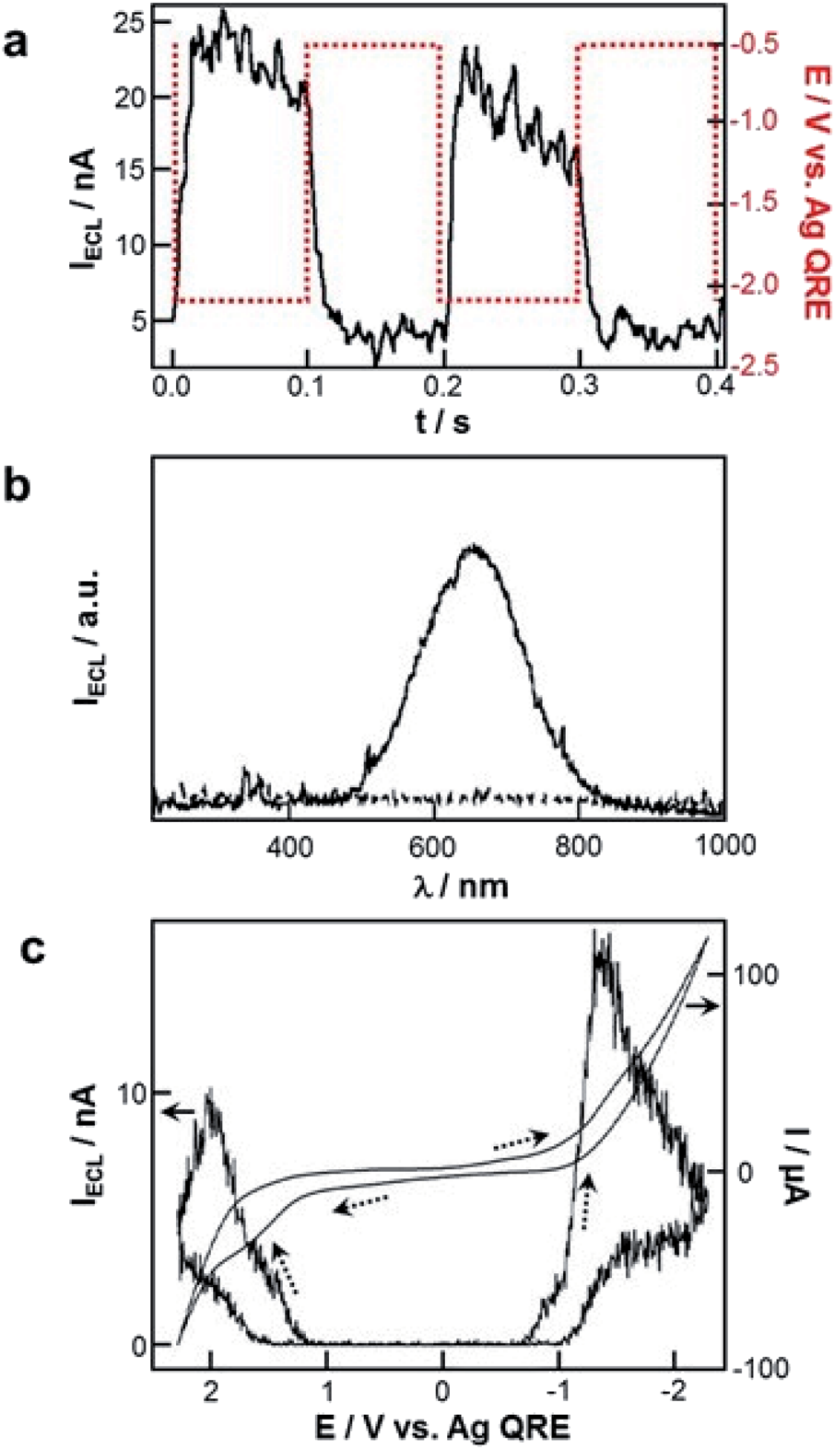
First ECL generation with QDs. (a) ECL transients recorded with Si nanocrystals and tetrabutylammonium persulfate co-reactant in DMF with tetrahexylammonium perchlorate as supporting electrolyte and (b) corresponding ECL spectra recorded with 10 Hz pulses for 10 min. Adapted from ref. 25, Copyright 2002 AAAS publications. (c) Cyclic voltammograms and ECL curves of CdSe nanocrystals in CH_2_Cl_2_ with tetrabutylammonium perchlorate as supporting electrolyte at a scan rate of 0.1 V s^−1^. Adapted from ref. 27 with permission from the American Chemical Society, Copyright 2002.

Several remarkable reviews summarizing the principle of ECL with QDs as well as potential applications such as bioanalysis and biochemical diagnostic were recently published.^19,30,185–189^ In this subsection, we will solely focus on the very first historical contribution in order to provide the reader a comprehensive view about when and how this field has emerged. In early 2002, Bard and co-workers reported the reversible electrochemical injection of discrete numbers of electrons into Si nanocrystals with a diameter of 2 to 4 nm.^25^ These Si particles were sterically stabilized with a capping agent consisting of a combination of hydrogen and alkoxide to afford good solubility in a variety of organic solvents such as DMF or acetonitrile. ECL was achieved following the annihilation pathway by pulsing the applied potential between +2.7 V and −2.1 V *vs.* Ag quasi-reference electrode at a frequency of 10 or 100 Hz. Also, the two possible co-reactant pathways were validated by using either oxalate or persulfate^190^ to achieve anodic or cathodic ECL, respectively (Fig. 9a). In all cases, the ECL spectra exhibited a peak maximum at ∼640 nm (Fig. 9b), which was significantly red-shifted by comparison with the corresponding PL spectrum (peak maximum at 420 nm when using an excitation wavelength of 360 nm). It is noteworthy that Bard and coworkers also investigated the ECL of Ge nanocrystals as a second example of elemental SCs.^26^ Compared to Si, Ge has a larger Bohr radius, which should lead to a more pronounced quantum confinement effect. These QDs were synthesized in a supercritical fluid by a growth-arrested process with octanol as a capping ligand. The average size was 4 nm with a rather heterogeneous dispersity. ECL transients were obtained in DMF by potential steps between +1.5 V and −2.5 V. The ECL signal was more intense during the oxidation pulses, suggesting that the reduced forms were more stable than the oxidized ones. The ECL spectra featured a maximum at ∼700 nm, which was again largely red-shifted compared to the PL spectra (peak maximum at 520 nm when using an excitation wavelength of 380 nm). This red shift, observed both with Si and Ge QDs can be readily explained by the involvement of surface states suggesting the key-role of surface passivation in the ECL emission. The reason is simply that charge injection events into nanocrystals should occur *via* surface states contrarily to PL that takes place deeper inside the nanocrystal. Therefore, completely passivated nanocrystals should lead to very similar ECL and PL spectra, which is not at all the case in these two reports.

Apart from these elemental QDs, ECL was also attempted with various metal chalcogenides. The first successful ECL candidate was CdSe nanocrystals that were synthesized by thermal treatment of Cd-acetate and Se powder precursors in presence of trioctylphosphine (TOP) and TOP-oxide (TOPO) as capping agents.^27^ The corresponding PL spectrum exhibited a maximum centered at 545 nm in chloroform when the solution was excited at 370 nm. The cyclic voltammograms collected in CH_2_Cl_2_ did not show very distinctive features compared to the supporting electrolyte alone but ECL was readily observed during the potential sweeps (Fig. 9c). Just like the previous report with Si QDs, the ECL spectra were red-shifted by ∼200 nm, suggesting once again that surface states are strongly involved in the ECL process.

Surprisingly, the same investigation performed with TOPO-capped CdTe in CH_2_Cl_2_, acetonitrile, and benzene revealed a very distinct behavior.^191^ First, the electrochemical characterization by cyclic voltammetry and differential pulse voltammetry revealed unambiguous electron transfer events. ECL signal was obtained only when scanning the potential toward the negative region, suggesting a very distinct lifetime of the electrogenerated species. Finally, the corresponding ECL spectrum was collected by pulsing the potential between −2.3 V and +2.3 V *vs.* Ag at 10 Hz with an integration time of 20 min. Although the ECL spectra were slightly broader than the PL ones, the corresponding maximum occurs at about the same wavelength (638 nm *vs.* 635 nm) without any significant red shift. The authors concluded that the as-synthesized CdTe nanocrystals did not have deep surface traps responsible for luminescence at a longer wavelength. Again, the model proposed to rationalize the ECL wavelength was that PL is dominated by excitation and emission within the core whereas electron-transfer events occur at the outer surface. Therefore, a direct proof of this model is to prepare highly passivated nanocrystals to prevent any redshift between the corresponding PL and ECL spectra. This was achieved with CdSe nanocrystals passivated with a shell of the wider bandgap material, ZnSe.^28^ Highly luminescent CdSe/ZnSe core/shell nanocrystals were prepared following a 2-step synthetic method consisting of the initial formation of CdSe seeds before a post-modification with a ZnSe shell. ECL properties were investigated in CH_2_Cl_2_ and the corresponding spectrum features a main peak at 580 nm, which is almost identical to the PL spectrum. This suggests that the accessible surface of the CdSe core was effectively passivated by the surrounding ZnSe core.

However, a careful examination of the ECL spectra revealed the presence of another weak and broad peak at ∼740 nm. The latter is indeed the signature of a non-quantitative passivation at the level of the dispersion of nanocrystals. This core/shell approach was not deeply investigated by the community working in the field of ECL. However, a very recent report suggested that the precise engineering of such structures enables a high level of control over the resulting ECL properties.^72^ The authors studied in detail a novel generation of QDs exhibiting hierarchical CdSe/CdS/ZnS core/shell/shell structures. Such a combination allows (i) enhancing massively the ECL efficiency as well as the signal stability, (ii) transferring the ECL capability from organic solvents to aqueous solutions and (iii) tuning the emission wavelength by varying the size of the QDs core. The ECL emission can be tuned from 549 nm up to 643 nm when increasing the average diameter from 5.9 to 9.0 nm, demonstrating the possibility to achieve spectrally resolved ECL measurements. Finally, the first report concerning CdS^192^ was documented about two years after the very first report due to the intrinsic instability of the corresponding electrogenerated species. The authors have not only tested cadmium sulfide but also engineered the shape of the QDs in order to enhance the ECL efficiency. In that context, they found that adjusting the shape of the QDs from spherical to hollow nanotubes by a stepwise nucleation and growth process afford a much more intense and stable ECL emission.^193^ Dual-color ECL based on PbS QDs and a BODIPY dye capping ligand was reported by Ding and coworkers.^194^ They obtained highly efficient ECL emission with this hybrid system using TPrA as a co-reactant. PbS QDs collisions with the electrode led to an enhancement of the ECL signal and it reached an efficiency of 96% relative to that of [Ru(bpy)_3_]^2+^, which is the highest among the SC QDs.^194^

### 5.3. From chemical to bioanalytical applications

As mentioned in the introduction, ECL is a very useful signal transduction technique that offers many possibilities for the development of analytical methods.^40^ In this subsection, we will summarize the principle approaches that were developed by using a QD as a luminophore.

#### 5.3.1. Detection of molecular targets

A first classic analytical strategy is to quantify the amount of ECL co-reactant whose concentration is proportional to the light emitted. As an example, ECL generation from CdTe nanocrystals was tested in aqueous solutions with a variety of amines as co-reactants.^195^ The advantage of amines relies on the application of only a mild potential, not exceeding 1 V to enable ECL. The authors compared the ECL efficiency depending on the nature of alkylamines. They found that the most efficient co-reactant is 2-(dibutylamino)ethanol (DBAE), which increases the ECL signal of about four times compared to TPrA. The corresponding calibration curve expressed in log scale has good linearity on five orders of magnitude with a detection limit (LOD) of a few nM.

**Fig. 10:**
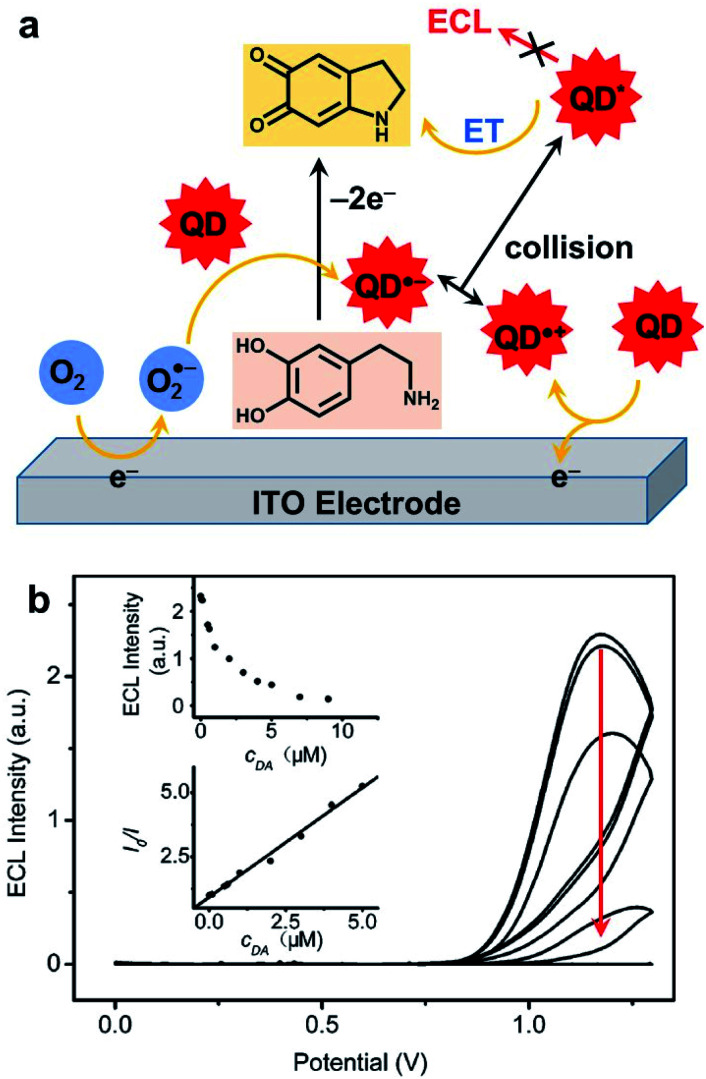
Detection of catechol with CdTe QDs. (a) Chemical mechanism leading to ECL quenching. (b) ECL curves collected in phosphate buffer without (top curve) and with an increase amount of dopamine (DA, from 50 nM to 50 μM) and figures of merit of the sensor (insets). Adapted from ref. 201 with permission from the American Chemical Society, Copyright 2007.

Hydrogen peroxide is also a classic reactive chemical that can be detected by ECL.^196^ The very first H_2_O_2_ sensor that take advantage of QDs ECL was based on a graphite electrode modified with CdSe nanocrystals in reduction in presence of molecular dioxygen.^197^ Interestingly, when the solution is deaerated with nitrogen, the signal becomes sensitive to H_2_O_2_ that can oxidize the reduced QD. The detection was proportional to H_2_O_2_ concentration in the micromolar range down to 100 nM. The combination of CdS nanocrystals with carbon nanotubes (CNTs) was also proposed in order to enhance ECL signal and lower the required overpotential.^198^ Using linear sweep voltammetry, the maximum ECL signal is collected at −1.2 V with CdS/CNT composite instead of −1.35 V for CdS alone. This strategy allows probing hydrogen peroxide up to the millimolar range. The precise molecular mechanism involved in these peroxide sensors was later rationalized with OH˙ radicals identified as key reactive species.^199^ The influence of the size of CdSe QDs on the ECL wavelength and strength was studied in detail. The smallest crystals with a diameter of 1.5 nm emitted at 396 nm but with poor efficiency. Increasing gradually the size up to 3.5 nm resulted in a red shift up to 570 nm. However, 2.5 nm in diameter was the best compromise with a maximum ECL efficiency calculated relative to the QD surface concentration. In a twist, the authors anticipated that radical scavengers might quench the ECL signal. This effect was validated by using thiol compounds. The quantitative detection of l-cysteine and glutathione (GSH) was successful in the micromolar range up to 60 μM with no effect of the corresponding disulfides (GSSG and l-cysteine, respectively). It is noteworthy that another comparable strategy in oxidation was also proposed for GSH detection.^200^ This time, the idea was to combine CdTe QDs with graphene oxide (GO) on an ITO electrode. GO facilitated the oxidation of these QDs and triggered the production of O_2_˙^−^ radical anion. The latter is readily reduced in presence of GSH, leading to ECL quenching in a typical off-signal sensor with a linear range from 20 to 200 μM. This quenching strategy has proved to be quite general since many redox-active targets can be addressed as evidenced by the detection of catechol derivatives.^201^ The ECL detection of H_2_O_2_ was also exemplified with QDs in a closed bipolar electrochemical configuration.^202^ In that case, two half cells with one feeder electrode are connected to a potentiostat while the bipolar electrode enables the electron transfer between both compartments. The authors selected perovskite-type CsPbBr_3_ as well as CdSe/ZnS QDs that afford a strong anodic ECL signal in presence of TPrA. The efficiency of both QDs was compared for H_2_O_2_ sensing but the perovskite offers a slightly lower LOD and linear range. A composite CdZnSeS QDs immobilized on an ordered mesoporous carbon substrate was also proposed for the detection of H_2_O_2_ in living cells.^203^ The ECL mechanism involves the simultaneous reduction of QDs and H_2_O_2_ target and OH˙ acts again as a key reactive intermediate that reacts with the QD inside the electrochemical reactivity layer. The calibration was linear from the millimolar down to the micromolar range and the practical use of this biosensor was validated by measuring H_2_O_2_ released from a variety of cell lines. Anodic ECL of CdTe QDs was used for the detection of dopamine and adrenalin on an ITO electrode. Again, the sensing principle involves the oxidation of the catechol to the corresponding quinone that suppresses the ECL emission through an intermolecular charge transfer (Fig. 10a). In this case, the calibration curve revealed a linear relationship between the catechol concentration with respect to the depletion of the ECL strength in the micromolar range (Fig. 10b). More recently, the same approach was proposed with CdS QDs with a tunable diameter, ranging from 1.8 to 3.7 nm.^204^ The size of these QDs directly affects the ECL wavelength as well as the onset potential necessary to drive cathodic ECL with persulfate. When using the largest QDs the quenching of ECL by dopamine was again used to propose an ultrasensitive ECL sensor with a wide linear detection range between 8 pM up to 20 nM.

#### 5.3.2. Enzymatic assays

The coupling of QDs ECL with enzymatic systems was demonstrated with two archetypical examples of cholesterol oxidase^205^ and acetylcholine esterase.^206^ A composite made out of CdS QDs and CNTs was initially developed for H_2_O_2_-driven ECL whose intensity was dependent on the presence of dioxygen in the solution. Enzyme-based biosensors were prepared by cross-linking these enzymes with glutaraldehyde on CNT-CdS modified electrodes for the detection of choline and acetylcholine, respectively. QDs are electrochemically reduced and drive the conversion of acetylcholine to choline prior to the oxidation of choline in presence of O_2_ with concomitant production of H_2_O_2_. The ECL signal in absence of substrates remains rather weak whereas it increases in presence of the enzymatic substrates. Following this strategy, choline can be detected in the micromolar range with a LOD of ∼1–2 μM whereas acetylcholine is monitored with a LOD of 2 to 5 μM.

Instead of detecting a given enzymatic substrate, a biosensor targeting directly small proteins can also be achieved.^207^ For instance, CdS nanocrystals were selected in combination with persulfate co-reactant, which affords a good cathodic ECL signal in aqueous solution. Apolipoprotein B (apoB-100) was covalently bound to the modified electrode in order to anchor a specific ligand to the targeted low-density lipoprotein (LDL). The stepwise preparation of the modified electrode was followed by using a variety of techniques such as electrochemical impedance spectroscopy and atomic force microscopy. The LDL target that hindered the ECL reaction efficiency was revealed by a decrease in ECL intensity upon specific binding to apoB-100. In this report, the ECL intensity of the biosensor inversely correlates with LDL concentration in the range from 25 pg mL^−1^ up to 16 ng mL^−1^ with a very low LOD of 6 pg mL^−1^. An ECL assay for alkaline phosphatase activity was also recently proposed.^208^ The substrate of this enzyme, which is a phosphate derivative of ascorbic acid is readily converted into ascorbic acid. The latter can directly act as a co-reactant to induce anodic ECL of CsPbBr_3_ QDs. A good linearity was found between the ECL intensity and the logarithm of alkaline phosphatase concentration with a LOD slightly lower than 1 mU per liter.

#### 5.3.3. DNA detection

Several contributors to the field also proposed the development of DNA-based ECL sensors. For example, the detection of thrombin with CdS nanocrystals was reported in cathodic ECL mode with persulfate.^209^ The QDs were immobilized and labeled with a specific aptamer that was conjugated with a DNA to capture Au NPs through hybridization. Such a configuration enables an efficient energy transfer between the Au NPs and the QDs, resulting in strong ECL. On the contrary, the recognition of thrombin by the aptamer releases Au NPs and suppresses the ECL signal. More sophisticated approaches that involve DNA and aptamers were also combined with DNA-cycle amplification in order to develop an ultrasensitive assay.^210^ This was applied to the detection of cancer cells with an analytical performance enabling the detection of several tens of cells per milliliter. More recently, a very innovative distance-mediated plasmon coupling between a CdS thin film and Au NP dimers was reported.^211^ CdS QDs were capped with a DNA probe enabling the immobilization of either one or two Au NPs at close proximity from each other. The distance between the electrode surface and the NPs was varied from 8 to 18 nm with different lengths of DNA duplex. The maximum ECL enhancement was monitored with an intermediate distance of 12 nm, enabling a 7-fold amplification. The wide bandgap of boron nitride (BN) can be regulated by sulfur containing dopants such as thiourea or cysteine, contributing to a wide tuning of the emission wavelength.^212^ These so-called S-regulated BN QDs were used to develop an original ratiometric ECL platform for gene detection. According to this essay, the recognition of the DNA target triggers the surface immobilization of Au NPs that will enable surface plasmon coupling. The ECL peak at 535 nm obtained with cysteine QDs remained unchanged whereas the ECL of the thiourea analogue at 620 nm is enhanced by the coupling affording a concentration-dependent readout down to 0.3 pmol L^−1^. Another smart DNA-mediated strategy based on Au–Au NPs dimer was developed recently.^213^ The hybridization of a target DNA by the complementary capture DNA triggers the immobilization of the Au dimer and subsequent surface plasmon coupling with carbon nitride QDs. Cathodic ECL with persulfate co-reactant was chosen as signal readout affording a wide dynamic range for sequence-specific DNA sensing. Surface plasmon coupling can also confer unusual properties to ECL^214^ emission as exemplified with Au nano-triangles.^215^ In this very recent report, these NPs were self-assembled at the electrode surface before ECL recording with SnS_2_ QDs. The pattern not only provides the signal enhancement but also generates an anisotropic circular polarization. This new property can be used for polarization-resolved DNA biosensing as exemplified with sensitive mRNA detection from 1 fM to 1 nM. A bipolar electrochemistry paper-based ECL assay was also reported for the detection of mRNA targets.^216^ The authors used two parallel channels where the cathodic pole of the bipolar electrode stripes is modified with either CdTe or g-C_3_N_4_ light-emitting probes. The selection of these two different QDs offers potential-resolved capability par parallel DNA screening. Also, amplification cycles performed in solution prior to surface immobilization affords an excellent LOD, which is typically in the fM range. Lead halide perovskites such as CsPbBr_3_ QDs were used in combination with a porous metal–organic framework to evaluate polynucleotide kinase activity and screen potential inhibitors.^217^ The enzyme activity triggers a DNA sequence of hybridization and surface immobilization that inhibits anodic ECL in presence of TPrA. A kinase detection limit of 6 μU mL^−1^ is achieved, which is more sensitive to any previous assay based on fluorescence or electrochemical readout. In the context of DNA sensing, a rarely reported elemental sulfur QD was used for ECL reporting that involves a DNA walking machine.^218^ Sublimed sulfur is aggregated with a PEG capping agent, prior to chemical etching with H_2_O_2_ to afford an intense and stable cathodic ECL emission in the near infrared (730 nm). ECL signal variation was found to depend on the flexibility of the DNA arm since the rigidity of a triple-stranded DNA scaffold provides a well-ordered track for the DNA walking machine.

#### 5.3.4. Immunosensing

ECL transduction is widely applied to biochemical diagnostic especially for immunosensing with a large number of tests run on a daily basis. In this context, several approaches that take benefit from QDs as a luminophore were proposed. ECL of CdSe QDs was used for the detection of human prealbumin (*i.e.*, PAB antigen).^29^ A layer-by-layer approach enables the immobilization of gold NPs and CdSe nanocrystals with a terminal layer featuring anti-PAB probes. The ECL mechanism involves persulfate whose diffusion towards the electrode surface is inhibited upon the formation of the immunocomplex with PAB target. Due to the highly specific recognition step, the sensor exhibits a wide dynamic range over four orders of magnitude with a LOD as low as 10 pg mL^−1^. The same group also exemplify the versatility of such an approach by developing another sensor for the detection of human immunoglobulin G (HIgG).^219^ The modified electrode was fabricated with a composite mixture of CNTs and chitosan polymer together with a silane ligation to anchor the antibody. As previously, the immunoreaction lowers the ECL efficiency and this detection is highly sensitive with a typical LOD of 1 pg mL^−1^. In another series of contributions, a novel strategy to enhance the band-gap of CdTe nanocrystals was proposed.^220^ These CdTe QDs were synthesized in aqueous media with a combination of stabilizers by refluxing the precursors for a minimum of 10 hours. Here, the key parameter was the refluxing time since the authors observed a progressive redshift of the PL and ECL spectra up to ∼800 nm. The ECL was observed by applying only a mild anodic potential in presence of TPrA. This finding opened the door to near-infrared ECL with these QDs that were employed in a sandwich-type immunoassay.^221^ α-Fetoprotein (AFP) antigen was recognized by a primary antibody immobilized at the surface of the electrode prior to secondary antibody capture. The latter was decorated with CdTe QDs to enable a positive ECL signal in the presence of DBAE. The sensor was successfully tested on a large dynamic range and was accurate down to an AFP concentration of 5 pg mL^−1^. Based on a comparable synthetic procedure, highly passivated ternary CdZnSe QDs were fabricated by the same group.^222^ This time, the sensor was operated in cathodic ECL mode with persulfate affording a detection of AFP down to 10 pg mL^−1^. The detection of chloramphenicol, which is a bacteriostatic broad-spectrum antibiotic was also achieved through an ECL-based immunoassay.^223^ The authors selected SnS_2_ QDs that were used in reduction with persulfate co-reactant. The ECL signal was enhanced by using a hollow TiO_2_ spherical shell that was involved in the ECL mechanism by intermolecular charge transfer with the QDs. Chloramphenicol antigen/antibody recognition enables a sensitive ECL signal to detect the chemical target below 10 ng mL^−1^ concentration.

**Fig. 11:**
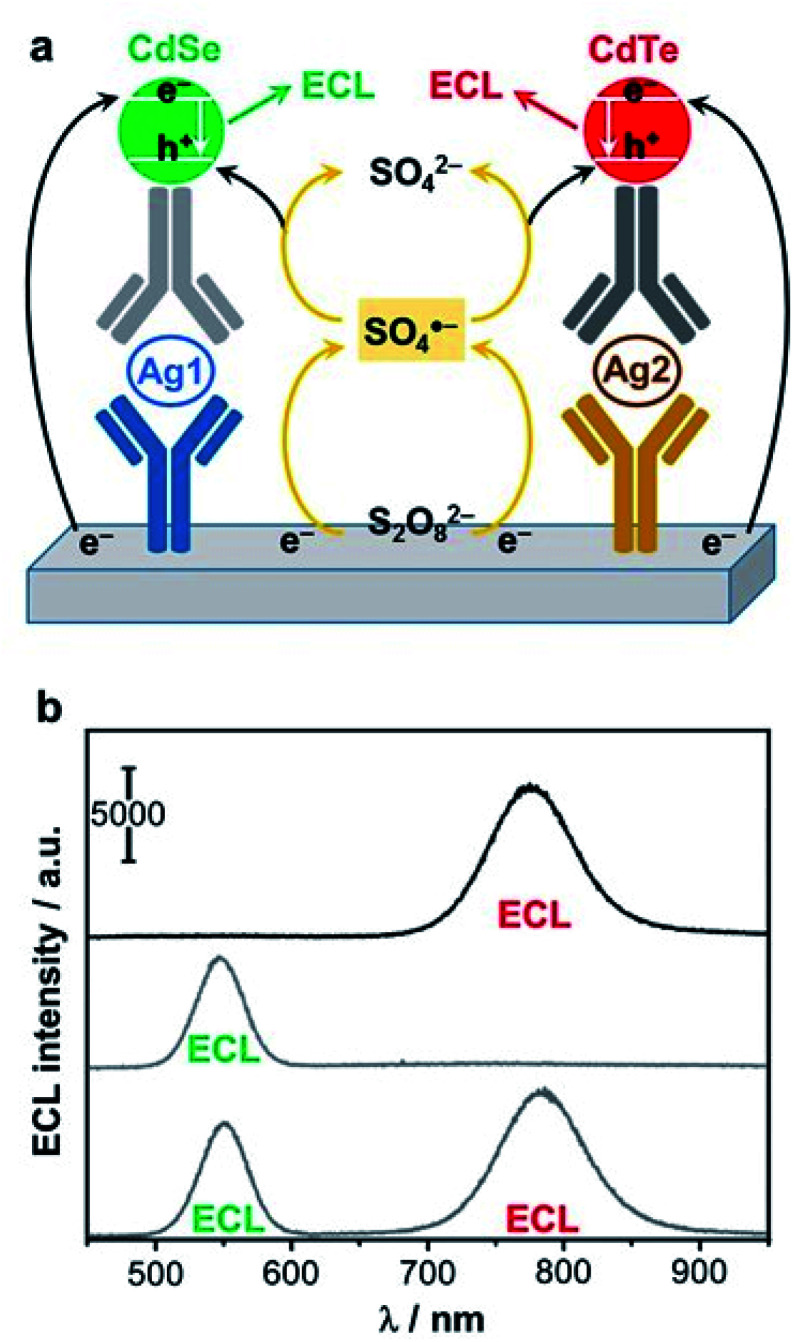
Spectral-resolved detection of two antigens (Ag1 and Ag2) with two different QDs. (a) Scheme of the cathodic ECL mechanism with persulfate co-reactant. CdSe QD is a green emitter (*λ*_max_ = 547 nm) whereas CdTe is a red emitter (*λ*_max_ = 783 nm). (b) ECL spectra for the detection of 100 pg mL^−1^ of α-fetoprotein (top), 10 ng mL^−1^ of carcinoembryonic antigen (middle) and simultaneous detection at the same targets concentration (bottom). Adapted from ref. 224 with permission from American Chemical Society, copyright 2017.

Finally, one of the most recent trends in the field of QD-based ECL sensing is to achieve multiplexing. This was made possible by employing simultaneously several QDs as proposed initially by the groups of G. Zou and W. Miao.^224^ The authors selected CdSe and CdTe QDs that emit at different wavelengths (*λ*_max_ = 550 nm *vs.* 776 nm) and fabricated a dual sandwich-type immunoassay (Fig. 11a) based on a cathodic ECL signal with persulfate. Practically, the biosensor can detect separately or simultaneously two targeted antigens, and the proof-of-principle was established with AFP and carcinoembryonic antigen (CEA) as models (Fig. 11b). The dual detection can be achieved with the same LOD that an individual sensing of the two antigen targets. Later, a triplex-color ECL multiplexing was also demonstrated by introducing a third CdTe QD that exhibits an intermediate wavelength of emission (*λ*_max_ = 650 nm).^225^ The later enables the simultaneous detection of the CEA, PSA and AFP. The same group also extended this approach for the simultaneous detection of two DNA targets.^226^ This was demonstrated with a biosensor that discriminates a wild type sequence-specific transcription factor (p53) from mutant p53 that are 21-mer oligonucleotides. Since both QDs do not exhibit the exact same efficiency, the choice of QD directly affects the dynamic range and LOD. For instance wild type p53 was detected down to 10 fM with CdTe QD (*λ*_max_ = 782 nm) whereas the LOD for mutant p53 was 5 fM with CdSe QD (*λ*_max_ = 554 nm).

## 6. Conclusions and outlooks

ECL has recently attracted an increasing attention due to its advantages such as, for example, high sensitivity, low background, miniaturization, simplicity of operation, high throughput analysis, and imaging capability. It is a very versatile technique because it is based on orthogonal principles in electrochemistry and photophysics. On the other hand, SC (nano)materials science is a very fast-moving research field with challenges in energy conversion, analytical chemistry, and photoelectrochemistry implying photocatalytic surface reactions upon light exposure. In this review, we show how ECL profits from the recent advances in SC photoelectrochemistry and material sciences. PECL has been demonstrated in various configurations. Both electrochemically assisted upconversion process (or “anti-Stokes PECL”) with the wavelength shift from red to blue and “Stokes PECL” have been demonstrated in aqueous environment opening new detection schemes in (bio)analytical chemistry. The stability of the SC surface and its passivation, especially of silicon used in the anodic regime in water, are important limiting issues that have been addressed with the deposition of nanometer-thick protective layers. In addition, a particularly attractive feature of SC is to spatially address the photoelectrode and to generate only locally the ECL emission.^174^ The spatiotemporal control of ECL generation by light offers the possibility to address microscopic photosensitive sites in a transient way. Another appealing possibility provided by SC photoelectrochemistry is the onset of ECL generated at unprecedented low anodic potentials for luminol, its L-012 derivative, and [Ru(bpy)_3_]^2+^.^111,174,179,180^ However, it is important to notice that other electrochemical (potentially interfering) reactions will be shifted to lower potentials as well in the PECL mode. Frontside and backside geometries have been reported for PECL. The backside configuration conjugated with “anti-Stokes” PECL features (*i.e.*, where the illumination light does not generate the fluorescence of the sample) may ensure that the SC electrode-liquid interface remains under complete dark conditions. This is particularly advantageous to avoid the photodegradation of sensitive biological samples and photobleaching of the ECL dye^179^ as well as to maintain extremely low the optical background signal of the ECL method. As future trends in this field, we can expect that ECL at SC electrodes will be employed to localize and quantify intrinsic phenomena related to SCs. Indeed, the SC surfaces often comprise regions of different natures such as crystallographic faces or defects that can induce different reactivity or different charge carrier dynamics locally.^227^ ECL, and particularly ECL microscopy can be a powerful tool to localize, *in situ*, active and inactive regions of a SC surface, and can be important in material engineering for improving the performance of SC materials (such as their stabilities) and also for localizing catalytic active sites, as SC materials can be employed as catalysts.

We also anticipate that PECL will be used as a powerful tool in the field of photoelectrocatalysis (*i.e.*, solar H_2_ production or CO_2_ valorization) as many photoelectrode architectures are made by inhomogeneous coatings. Therefore, visualizing precisely the location where energy conversion occurs (*e.g.*, hole transfer at a nanoparticle immobilized on a SC for oxygen evolution reaction)^109^ is a great challenge and has the potential to trigger considerable advance in the field of solar fuels. Further, PECL can be employed as an interesting tool for IR conversion and thermal imaging. In this case, it is important to develop photoelectrodes allowing at the same time intense visible emission and high resolution. While progress has been made recently in SC protection, providing a SC photoelectrode able to generate anti-Stokes PECL for several days is still a bottleneck that prohibits realistic applications. Cathodic PECL may provide a solution to this problem in a short term perspective, because several narrow-band-gap semiconductors are relatively stable in the cathodic regime. Other important aspects are the choice of the ECL system, the selection of the SCs and their (micro/nano)-structuration, and the design of the optical/electrochemical setup, which offer new degrees of freedom (*i.e.*, more tools, techniques, and concepts in the ECL toolbox) and versatility for the ECL technology. From a practical point of view, the different forms of PECL combined with the continuous boom in SC industry permit various ECL applications at low cost with disposable devices or imaging systems. We therefore expect that PECL will undergo a rapid further development in the near future and lead to promising new (bio)analytical and imaging applications.

Instead of using a SC as the electrode material in the ECL field, their nanocrystal forms or QDs have attracted considerable research interest as ECL emitters. Indeed, they are bright ECL nano-luminophores with tunable emission wavelengths and exceptional photo- and chemical stability. The advances in the fabrication process of these NPs formulated as colloidal suspensions provide remarkable control over their dimension, dispersity, and shape. It is essential because their shape, surface, and coating determine their optical and electronic properties. Depending on the QDs structures and surface chemistry, the chemical reactivity of QDs can result in either disastrous or remarkable performances. Since the ECL generation of the QDs excited state follows a different mechanism in comparison to PL or electroluminescence, the design of ECL-active QDs should fulfill specific criteria to avoid surface traps of the charge carriers. It is essential to design judiciously the bandgap and lattice structure of QDs for ECL applications. This can be achieved by using strongly protective ligands or additional shells (*e.g.*, ZnS) with a very wide bandgap. These approaches provide an enhanced barrier to isolate those surface traps accessible for ECL generation. New synthetic methods offer the possibility to engineer the interior inorganic structure with core/shell/shell structures and inorganic–organic interfaces. Therefore, the detailed comprehension of all these parameters (*i.e.*, structure-property relationships) and interfaces is essential to develop highly efficient water-soluble ECL QDs with multiplexing capabilities. The tailored and rational design and fabrication of QDs specifically for ECL are thus necessary. Then, the synthesized bright and stable nano-emitters can find diverse applications in multiplexed bioassays, ECL resonance energy transfer detection schemes and ECL microscopy. Since metal-based QDs are toxic, it is important to use low-toxicity, eco-friendly alternatives to develop QD-ECL emitters. Undoubtedly, the versatility offered by the combination of ECL with SC (nano)materials opens a wide range of opportunities to develop new ultrasensitive (bio)sensing and imaging applications, especially in point-of-care testing, confined and multiphase systems,^178,228,229^ biological assays, and microscopy of cellular and subcellular structures.

## Author contributions

N. S. and G. L. defined the scope and the structure of the review article. All the authors prepared the manuscript by writing the initial draft, drawing or selecting the illustrations, reviewing and editing.

## Conflicts of interest

There are no conflicts to declare.

## Supplementary Material
